# Local Adaptation in European Firs Assessed through Extensive Sampling across Altitudinal Gradients in Southern Europe

**DOI:** 10.1371/journal.pone.0158216

**Published:** 2016-07-08

**Authors:** Louise Brousseau, Dragos Postolache, Martin Lascoux, Andreas D. Drouzas, Thomas Källman, Cristina Leonarduzzi, Sascha Liepelt, Andrea Piotti, Flaviu Popescu, Anna M. Roschanski, Peter Zhelev, Bruno Fady, Giovanni Giuseppe Vendramin

**Affiliations:** 1 INRA, UR629 URFM Ecologie des Forêts Méditerranéennes, Domaine Saint Paul, Site Agroparc CS 40509, 84914 Avignon Cedex 9, France; 2 Institute of Biosciences and BioResources, National Research Council (IBBR-CNR), Division of Florence, Via Madonna del Piano 10, 50019 Sesto Fiorentino (FI), Italy; 3 Scuola Superiore Sant'Anna, Piazza Martiri della Libertà 33, 56127 Pisa, Italy; 4 National Institute of Forest Research and Development (INCDS), Research Station Simeria, Str. Biscaria 1, 335900 Simeria, Romania; 5 Department of Ecology and Genetics, Evolutionary Biology Center and Science for Life Laboratory, Uppsala University, 75236 Uppsala, Sweden; 6 School of Biology, Aristotle University of Thessaloniki, GR-54124, Thessaloniki, Greece; 7 Institute of Biosciences and BioResources, National Research Council (IBBR-CNR), Division of Palermo, National 3. Research Council—Corso Calatafimi, 414—I-90129, Palermo (PA), Italy; 8 University of Marburg, Faculty of Biology, Conservation Biology, Karl-von-Frisch-Straße 35032 Marburg, Germany; 9 Leibniz Institute of Plant Genetics and Crop Plant Research (IPK), Genebank Collections North, Inselstrasse 9, D-23999 Malchow/Poel, Germany; 10 University of Forestry, 10, Kl. Ohridsky Blvd., 1797 Sofia, Bulgaria; Umeå Plant Science Centre, Umeå University, SWEDEN

## Abstract

**Background:**

Local adaptation is a key driver of phenotypic and genetic divergence at loci responsible for adaptive traits variations in forest tree populations. Its experimental assessment requires rigorous sampling strategies such as those involving population pairs replicated across broad spatial scales.

**Methods:**

A hierarchical Bayesian model of selection (HBM) that explicitly considers both the replication of the environmental contrast and the hierarchical genetic structure among replicated study sites is introduced. Its power was assessed through simulations and compared to classical ‘within-site’ approaches (FDIST, BAYESCAN) and a simplified, within-site, version of the model introduced here (SBM).

**Results:**

HBM demonstrates that hierarchical approaches are very powerful to detect replicated patterns of adaptive divergence with low false-discovery (*FDR*) and false-non-discovery (*FNR*) rates compared to the analysis of different sites separately through within-site approaches. The hypothesis of local adaptation to altitude was further addressed by analyzing replicated *Abies alba* population pairs (low and high elevations) across the species’ southern distribution range, where the effects of climatic selection are expected to be the strongest. For comparison, a single population pair from the closely related species *A*. *cephalonica* was also analyzed. The hierarchical model did not detect any pattern of adaptive divergence to altitude replicated in the different study sites. Instead, idiosyncratic patterns of local adaptation among sites were detected by within-site approaches.

**Conclusion:**

Hierarchical approaches may miss idiosyncratic patterns of adaptation among sites, and we strongly recommend the use of both hierarchical (multi-site) and classical (within-site) approaches when addressing the question of adaptation across broad spatial scales.

## Introduction

Local adaptation is the evolutionary process by which populations diverge toward different phenotypic and genetic optima in response to their local ecological conditions. Forest trees provide numerous examples of adaptive divergence across a variety of spatial scales, and local adaptation is supposedly a key process of tree populations’ evolution and species’ diversification [1]. Indeed, forest tree species are often widely distributed across sharply contrasted environmental conditions, and local adaptation is favored in trees as a result of large population sizes and high levels of genetic variation for fitness-related traits [2–12]. In particular, climate is one of the most important drivers of adaptation in forest tree populations [1, 10, 11, 13–18] and understanding the molecular bases of adaptation to climatic conditions is essential to accurately predict trees’ responses to global climate change (GCC, [19]). European forests are facing enormous threats from rapid GCC with increasing frequency and intensity of summer drought, while considerable uncertainties exist about plants potential to respond to future warming and declining moisture availability [20]. Although recent evidence suggests that pollen flow can connect populations more than 10^2^−10^3^ km apart [21], escaping GCC through migration will also require substantial seed dispersal, which can be a limiting factor for many tree species. Those populations will thus have to cope locally with environmental changes, through individual physiological tolerance (*i*.*e*. phenotypic plasticity) in a proximate time (a time period corresponding to the individual lifespan), and through evolutionary change (*i*.*e*. genetic adaptation over generations, [22, 23]). Notwithstanding large genetic variation and potentially fast adaptation, there is only anecdotal evidence that forest trees can genetically adapt to contemporary environmental change over a limited number of generations. In this context, studying species at the limits of their distribution range is particularly important for predicting the future evolution of species and their peripheral populations [10].

Outlier methods (*i*.*e*. *F*_*ST*_-based outlier detection tests) are frequently used to study local adaptation by identifying loci showing strong differentiation across populations [13, 24]. Their basic rationale is that loci influenced by natural selection toward different genetic optima across populations (*i*.*e*. divergent selection) are expected to be more differentiated than neutral loci, while loci subject to selection toward the same optimum across populations (*i*.*e*. homogenizing selection) are expected to be less differentiated than neutral ones [25–29]. Despite its conceptual simplicity, the *F*_*ST*_–outlier approach suffers from several limitations, *e*.*g*. its sensitivity to heterogeneity in demographic histories among populations and lineages [30, 31], subsequent complex spatial structuring [27, 32, 33] and variations in recombination and linked selection across the genome [34]. To overcome these problems, it is recommended to apply different methods to reduce the risk of false positives [35–37] and to use replicated population comparisons [31, 32]. Two approaches are widely used to identify outliers under selection: the coalescent approach (FDIST) by Beaumont and Nichols [26] and the Bayesian method (BAYESCAN) by Foll and Gaggiotti [28]. In particular, Bayesian modelling is very powerful to empirically calibrate complex models without *a priori* assumptions about the magnitude of parameters to infer [38]. This approach has revolutionized the field of population genetics [39, 40] and is now commonly used to analyze the genetic structure of populations [41–43] and to identify outliers for selection [28]. Moreover, the Bayesian *F*_*ST*_-outlier selection test developed by Foll & Gaggiotti [28] has been shown to generally result in lower type I and II error rates than the original coalescent method by Beaumont and Nichols [26].

Although candidate gene approach has proved to be effective in detecting adaptive genetic differentiation in species with large genomes [18, 44–47], only few studies attempted to detect divergent selection to altitude using replicated population pairs spread across large spatial scales. Indeed, developing *ad hoc* models able to analyze replicated population pairs [31] is necessary to address the question of local adaptation in natural settings using replicated sampling designs. This is important since failing to account for demographic history [30, 48] and, more generally, for hierarchical genetic structure [27, 36, 49, 50] can lead to a lack of detection power or to the detection of false positives when analyzing replicated populations pairs simultaneously. It is thus necessary to develop methods adapted to large datasets and able to reduce the detection of false positives [27, 32, 36, 51–56].

The main objective of this work was to identify molecular evidence of adaptation to altitude in a conifer tree, *Abies alba* Mill., by sampling replicated populations pairs at study sites spread across its southern distribution range, where populations are expected to face strong selective pressures [10, 57]. To this end, a Bayesian model that explicitly considers both the replication of the environmental contrast ‘low *versus* high’ elevation and the hierarchical structure among replicated sites was developed. Pairs of populations were sampled at nine *A*. *alba* study sites plus one additional site of the congeneric species *A*. *cephalonica* Loudon. About 1600 individuals were genotyped using 273 SNPs within expressed sequences (ESTs), with the aim of deciphering the genetic outcomes of local selection in a keystone European forest tree species.

## Material and Methods

### Ethics statement

Adult trees of *A*. *alba* were sampled in nine study sites in France, Italy, Bulgaria, and Romania (sites n°1 to 9). In France (sites n°1 to 5), no specific permission was required. Leaf sampling was nondestructive and carried out on public forest lands. In Italy (sites n°6 and 7), the sampling was authorized by the 'Ente Parco Nazionale del Gran Sasso e Monti della Laga'. In Bulgaria (site n°8), the sampling was carried out with permission and under the supervision of the ‘Pirin National Park Administration’. In Romania (site n°9), the sampling was carried out with permission from the private owner of the forest site.

In addition, one natural population of *A*. *cephalonica* was sampled in the Peloponnese, Greece (site n°10). The permission was given by the General Directorate for the Protection and development of Forests and Rural Environment of the Greek Ministry for Environment and Energy.

Field studies did not involve endangered or protected species.

### 1. Biological model

Silver fir (*A*. *alba* Mill., Pinaceae) is a widespread European conifer occurring in mountainous regions of central and southern Europe, usually between 500 and 1500 meters [58]. It is a keystone species of mountain forests with high ecological and economic value. *A*. *alba* populations belong to at least three main lineages (Pyrenees, Apennines and Balkan) that probably diverged during the Pleistocene as they remained at least partly isolated in multiple glacial refugia during Quaternary climatic cycles, including the last glacial maximum [59]. In addition, adaptive variations have already been reported in this species for quantitative traits, isozymes, and candidate genes [18, 60–63].

An additional study site of its congeneric species, Greek fir (*A*. *cephalonica* Loudon, [64]) was included in this study and treated separately from *A*. *alba* study sites to test whether local adaptation may be shared by two congeneric species. Greek fir is an endemic fir species widespread in the mountains of central and southern Greece from 400 to 1800 meters. Despite slight genetic divergence between the two fir species, they are closely related and able to produce fertile hybrids [59, 65, 66].

### 2. Sampling strategy and DNA extraction

**Fig 1:**
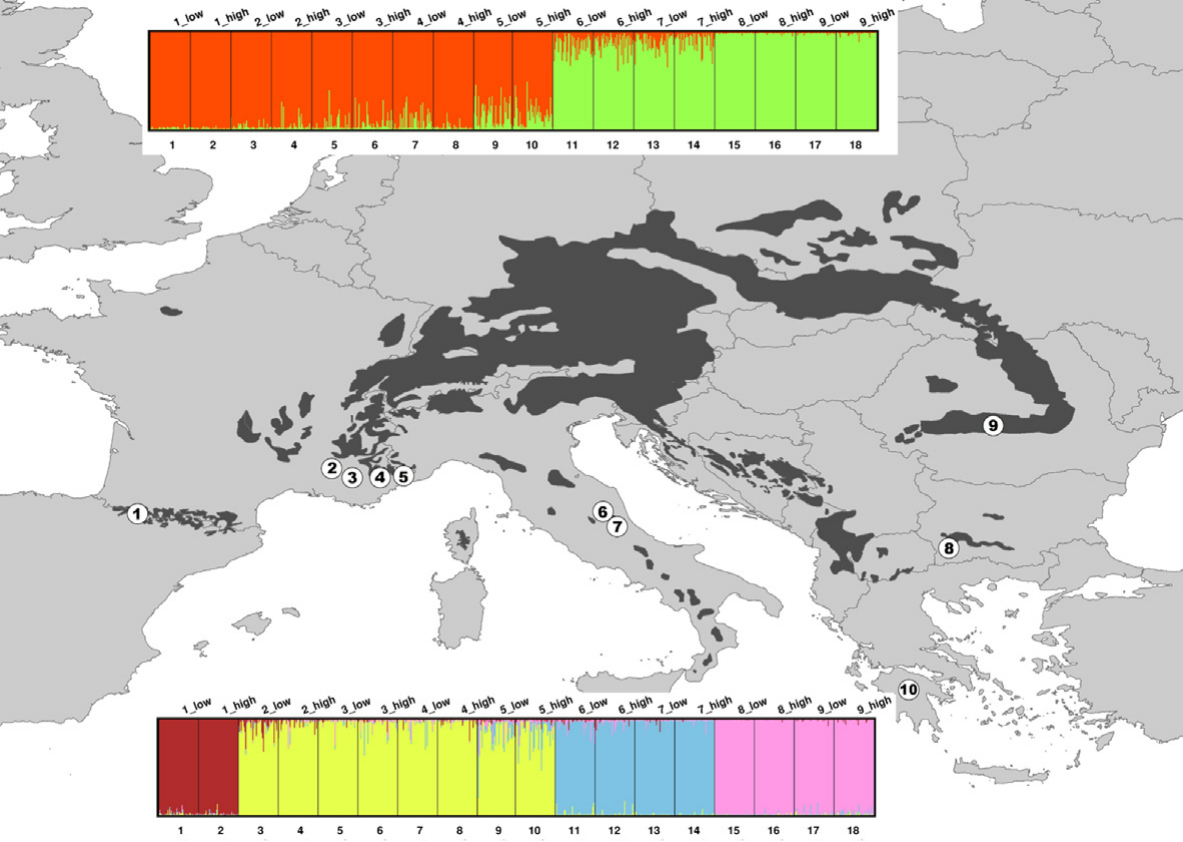
Genetic structure. Location of the study sites (*A*. *alba*, sites 1 to 9 and *A*. *cephalonica*, site 10) and genetic structure of *A*. *alba* populations revealed by STRUCTURE for *K* = 2 (top) and *K* = 4 (bottom). The map was created in ArcMap v.9.3 (ESRI. Redlands, CA). The European basemap is copyrighted by EUROSTATS (EuroGeographics for the administrative boundaries) and is available at: http://ec.europa.eu/eurostat/web/gisco/geodata/reference-data/administrative-units-statistical-units. The black area shows the distribution range of *A*. *alba* (according to EUFORGEN 2009, http://www.euforgen.org). Study sites IDs are described in Table 1.

Adult trees of *A*. *alba* were sampled in nine study sites located along the southern edge of the species distribution range, from the French Pyrenees to Romania (Fig 1, sites n°1 to 9), at two discrete elevations within each site (‘low’ versus ‘high’). One natural population of *A*. *cephalonica* located in the Peloponnese (Greece) was sampled according to the same strategy (site n°10). The location of the study sites and the sampling scheme are presented in Table 1. Material from the four French Alps sites is the same as in Roschanski *et al*. [62]. Genomic DNA was extracted by LGC Genomics (Middlesex, United Kingdom).

**Table 1:** *A*. *alba* and *A*. *cephalonica* study sites and sampling design.

					Number of samples		Elevation (meters)	
Study site ID	Country	Study site	Latitude (decimal degrees) WGS84	Longitude (decimal degrees) WGS84	low elevation	high elevation	low elevation	high elevation
***Abies alba* Mill.**								
1	France	Ossau Valley (Pyrenees)	42.855	-0.457778	81	82	825	1562
2	France	Ventoux (Alps)	44.17511	5.2437	249	290	995	1340
3	France	Lure (Alps)	44.11422	5.83912	55	56	1410	1628
4	France	Issole (Alps)	44.0242	6.46244	49	47	1108	1585
5	France	Vesubie (Alps)	43.97074	7.36577	43	45	1078	1497
6	Italy	Valle della Corte (Apennines)	42.70347	13.37576	48	48	1325	1580
7	Italy	Colle dell’Abete (Apennines)	42.66772	13.42677	47	47	1375	1600
8	Bulgaria	Bansko (Pirin Mountains)	41.843055	23.3852	48	48	1175	1750
9	Romania	Arges (Fagaras Mountains)	45.4411	24.6947	95	94	1070	1410
***Abies cephalonica* Loudon**								
10	Greece	Menalo Mt (Peloponnese)	37.68333	22.20639	48	48	1130	1525
**TOTAL number of samples = 1568**					**763**	**805**		

### 3. SNP design, genotyping, blastX and functional annotation

A total of 763 SNPs and surrounding sequences derived from a transcriptome assembly [67] were sent to LGC Genomics (Middlesex, UK) for KASP assay design. Candidate genes were selected based on a specific annotation procedure described in details by Roschanski *et al*. (2013) [67]. This set of SNPs has further been enriched by including transcripts related to drought based on gene ontology as described in Roschanski *et al*. (2016) [62] (for more details see these two related articles). Among the 763 SNPs tested for genotyping in the present study, 273 SNPs located within 177 transcripts were found to be amplifiable and polymorphic. These 273 SNPs were thus selected to genotype samples from the nine *A*. *alba* sites (n°1 to 9) plus the unique *A*. *cephalonica* site (n°10). Due to technical reasons, however, only 243 SNPs were successfully genotyped for the silver fir site of the Pirin Mountains (site 8, Bulgaria) and for the Greek fir site of Menalo Mt (site 10, Greece).

After genotyping, a standardized (with uniform parameters) and deeper (including KEGG pathways and enzyme codes) annotation of the targeted transcripts was realized. For this, the targeted transcripts were blasted (blastX) and annotated using Blast2Go software [68] with minimum e-value = 10^−6^, annotation cut-off = 55 and GO Weight = 5. This extended functional annotation was consistent with the hypothesis of climatic adaptation for a majority of them, confirming thus that the targeted transcripts can be reasonably considered as candidates for selection. This dataset is thus a non-random genome-scan enriched in candidate genes which is more relevant than classical genome-scans [69] because SNPs located in expressed candidates are more likely to experience selection than SNPs randomly sampled from the entire genome.

SNP design, blastx, functional annotation and genotyping are provided on Figshare doi: 10.6084/m9.figshare.1418061 (https://figshare.com/articles/DATA_Abies_data/1418061).

### 4. Genetic structure analysis

Genetic structure was analyzed using STRUCTURE version 2.3 [42, 70]. Five independent runs for each *K* value ranging from 1 to 20 were performed under the admixture model, with a burn-in period of 15,000 followed by 25,000 iterations. The rate of change in *L(K)* across successive *K* values (*ΔK*) was calculated following Evanno *et al*. [71] using the web application ‘STRUCTURE Harvester’ [72].

### 5. Bayesian modelling, step 1: Bayesian allelic frequencies inference and G_ST_ estimation

The Bayesian modelling approach is composed of two independent steps. The first step aims at inferring allelic frequencies and estimating pairwise *G*_*ST*_, while the second step aims at partitioning the *G*_*ST*_ inferred through the first step into genome-wide and locus-specific parameters.

Allelic frequencies (*p*_*i*,*m*_ and *q*_*i*,*m*_) at each bi-allelic marker (*m*) within each population *i* (i.e. each *site×elevation* combination) were inferred independently for each marker and population from allelic counts (*n*_*i*,*m*_ and *N*_*i*,*m*_) through the equations:

$$\begin{array}{l} N_{i,m}∼Bin(p_{i,m},N_{i,m})\\ q_{i,m}=1-p_{i,m} \end{array}$$

The number of observations of a given allele (*n*_*i*,*m*_) within a population *i* at a bi-allelic marker *m* is sampled from a binomial distribution with parameters *p*_*i*,*m*_ (which is the allelic frequency in population *i* at marker *m*) and *N*_*i*,*m*_ (which is the total number of allelic counts in population *i* at marker *m*). A Beta distribution was chosen as prior for allelic frequencies *p*_*i*,*m*_: *p*_*i*,*m*_*~β(1*,*1)*.

In order to take into account uncertainties about allelic frequencies, pairwise *G*_*ST*_ between populations (*i*,*j*) for each marker (*m*) were estimated within the model, using Nei’s fixation index [73]:

$$G_{ST(i,j),m}=(H_{T(i,j),m}-H_{S(i,j),m})/H_{T(i,j),m}$$

with

$$H_{T(i,j),m}=\frac{1}{2}(p_{i,m}+p_{j,m})(q_{i,m}+q_{j,m})$$

and

$$H_{S(i,j),m}=(p_{i,m}q_{i,m})+(p_{j,m}q_{j,m})$$

The model was written in BUGS [74]. The code is available on Figshare doi: 10.6084/m9.figshare.1385214 (https://figshare.com/articles/Bayesian_GST_inference_BUGS_encoded/1385214).

### 6. Bayesian modelling, step 2: Bayesian hierarchical outlier detection (HBM = Hierarchical Bayesian Model)

Because our sampling design consisted of 9 replicates of local elevations spread across a broad geographical scale, we designed a hierarchical Bayesian model (hereafter ‘HBM’) to partition pairwise *G*_*ST*_ into genome-wide and locus-specific components under a two-level hierarchical model.

The median of *G*_*ST*_ inferred through the first step was used as input in the second step. A logit transformation was applied to *G*_*ST*_ values and a classical linear model was used to partition transformed *G*_*ST*_ into genome-wide effects corresponding to the different levels of neutral genetic structuring (clusters, sub-clusters within clusters and elevation), and into locus-specific effects related to elevation:

$$\begin{array}{l} \text{log}\;it(G_{ST(i,j),m})∼N(mean_{(i,j),m},\tau_{R})\\ mean_{(i,j),m}=\mu_{G}+(k_{Clus(i,j)}\mu_{Clus})+(k_{SubClus(i,j)}\mu_{SubClus})+(k_{Elev(i,j)}(\mu_{Elev}+\theta_{Elev(m)})) \end{array}$$

where

*k*_*Clus(i*,*j)*_, *k*_*SubClus(i*,*j)*_ and *k*_*Elev(i*,*j)*_ are binary matrices describing whether the two populations (*i*,*j*) belong to different clusters, to different sub-clusters within a same cluster, or to different elevations:

$$k_{Clus(i,j)}=\left(\begin{array}{c} 0\\ 1 \end{array}\right)$$

*k*_*Clus(i*,*j)*_ = 1 if the two populations (*i*,*j*) belong to different clusters, *k*_*Clus(i*,*j)*_ = 0 otherwise;

$$k_{SubClus(i,j)}=\left(\begin{array}{c} 0\\ 1 \end{array}\right)$$

*k*_*SubClus(i*,*j)*_ = 1 if the two populations (*i*,*j*) belong to different sub-clusters within the same cluster, *k*_*SubClus(i*,*j)*_ = 0 otherwise;

$$k_{Elev(i,j)}=\left(\begin{array}{c} 0\\ 1 \end{array}\right)$$

*k*_*Elev(i*,*j)*_ = 1 if the two populations (*i*,*j*) inhabit different elevation classes (low or high elevation), *k*_*Elev(i*,*j)*_ = 0 otherwise.

*μ*_*G*_, *μ*_*Clus*_, *μ*_*Subclus*_ and *μ*_*Elev*_ are genome-wide parameters describing the extent of genome-wide differentiation between clusters, sub-clusters within clusters and between elevations. *μ*_*G*_ is a global mean of differentiation that is inferred from all population pairs and captures the global extent of differentiation among all population pairs. *μ*_*Clus*_ captures the genome-wide effect of belonging to different clusters, *μ*_*SubClus*_ the genome-wide effect of belonging to different sub-clusters within a same cluster, and *μ*_*Elev*_ the genome-wide effect of belonging to different elevations (whatever the cluster or sub-cluster).

*θ*_*Elev(m)*_ are locus-specific parameters describing the extent of locus-specific genetic differentiation among populations belonging to different elevations. These parameters capture locus-specific effects of differentiation caused by elevation, assuming a common effect of elevation in all study sites whatever the cluster and sub-cluster.

Genome-wide and locus-specific parameters had non-informative priors:

*τ*_*R*,_ is the residual precision (*i*.*e*. inverse of the residual variance *1/σ²*_*R*_).

$$\mu_{G}∼N(0,\tau_{G})$$

with precision (*i*.*e*. inverse of the variance) *τ*_*G*_ = 0.0001, meaning:

$$\sigma_{G}^{2}=\frac{1}{\tau_{G}}=\frac{1}{0.0001}=10000$$

$$\mu_{Clus}∼N(0,\tau_{Clus})$$

$$\mu_{SubClus}∼N(0,\tau_{SubClus})$$

$$\mu_{Elev}∼N(0,\tau_{Elev})$$

The parameters *μ*_*Clus*_, *μ*_*SubClus*_, and *μ*_*Elev*_ are parameterized by a precision (*τ = 1/σ²*), following a Gamma distribution:

$$\begin{array}{l} \tau_{Clus}∼Gamma(0.01,0.01)\\ \tau_{SubClus}∼Gamma(0.01,0.01)\\ \tau_{Elev}∼Gamma(0.01,0.01) \end{array}$$

Similarly, the residual precision follow a Gamma distribution:

$$\tau_{R}∼Gamma(0.01,0.01)$$

At last, locus-specific parameters follow a normal distribution:

$$\theta_{Elev(m)}∼N(0,\tau_{m})$$

with precision *τ*_*m*_ = 0.0001 (*i*.*e*. *σ²*_*m*_ = 10000).

To make the model identifiable, the constraint Σ*θ*_*Elev(m)*_ = 0 was applied to the estimation of *θ*_*Elev(m)*_ parameters. *θ*_*Elev(m)*_ were sorted in decreasing order. Outliers were detected by fitting *a posteriori* a normal law from the inferred distribution of *ordered(θ*_*Elev(m)*_*)* and by attributing a probability of being under selection to each marker. Outliers on the upper-tail of the distribution are significantly more differentiated than expected under neutrality (*i*.*e*. above the neutral background), while outliers on the lower tail are significantly less differentiated than expected under neutrality (*i*.*e*. below the neutral background). Outliers above the neutral background are thus good candidates for divergent selection between environmental contrasts repeated among the different study sites. Outliers below the neutral background may have different origins: they may alternatively reflect equilibrium allelic frequencies between elevations resulting from homogenizing selection, or rare alleles in one or few study sites caused by purifying selection or other neutral evolutionary processes.

The code is available on Figshare doi: 10.6084/m9.figshare.1385218 (https://figshare.com/articles/HBM_Hierarchical_GST_partitioning_and_Outlier_detection_BUGS_encoded_/1385218).

#### 6.1. Running HBM on simulated data and empirical assessment of false-discovery rate (*FDR*) and false non-discovery rate (*FNR*)

To assess the statistical power and to explore the limits of our Bayesian modelling approach, HBM was applied to data simulated under different selection scenarios. Simulations were performed using the software SIMUPOP [75], an individual-based forward simulation of population evolution in Python language (http://simupop.sourceforge.net/Main/HomePage), S1 Method and Figshare doi: 10.6084/m9.figshare.1385219 (https://figshare.com/articles/Populations_simulation/1385219).

The statistical power of HBM to detect divergent selection in the replicated population pairs was assessed through simulated datasets composed of 16 populations of N = 200 diploid individuals each (totaling N = 3200 diploid individuals) under a hierarchical model of migration, by varying both the number of selected loci and the selection strength (*s = 1-ω* with *ω* the fitness, S1 Method).

Two thousand iterations with a burn-in of 1000 were largely sufficient to reach chain convergence and to obtain informative posteriors. The model was thus applied to simulated populations with 1, 5 or 10 loci (out of 100) under divergent selection of different strengths (uniform among loci): *s* = 0.05 (weak selection), *s* = 0.075 (moderate selection), *s* = 0.1 (strong selection), *s* = 0.5 and *s* = 0.99 (very strong selection leading to differential allele fixation at different elevations). The model was also applied to simulated data composed of 10 loci under divergent selection with variable selection strengths (*s*) among loci: 0.06 to 0.15, 0.15 to 0.25 and 0.06 to 0.25. Finally, the power of the model was investigated in the cases of homogenizing selection and of a combination of homogenizing and divergent selection. In the former case, populations with 5 loci under divergent selection with selection strength of 0.1 were simulated. In the latter case, populations with 2 loci under divergent selection and 2 loci under homogenizing selection with selection strength of 0.1 were simulated.

False-discovery rate (*FDR*) and false non-discovery rate (*FNR*) were empirically assessed [76, 77]: *FDR = V/R* where *V* is the number of false positives and *R* the number of rejected null-hypotheses, and *FNR = T/(m-R)* where *T* is the number of false negatives and *(m-R)* the number of accepted null hypotheses.

#### 6.2. Running HBM on *A*. *alba* data

Each *A*. *alba* population was assigned to a particular cluster and sub-cluster according to the structuring detected using STRUCTURE (for *K* = 2 and *K* = 4, see results section, S1B Fig), and the model was applied to the whole *A*. *alba* dataset using 10000 iterations with a burn-in period of 5000 iterations. The SNPs that were not genotyped in the nine *A*. *alba* study sites were discarded, resulting in a dataset composed of 243 SNPs successfully genotyped in all study sites (as described in section 3 ‘SNP design & genotyping’).

### 7. Within-site outlier detection tests (FDIST, BAYESCAN and SBM)

Because the Bayesian model was developed to find replicated evidence of divergent selection in replicated population pairs, it was inherently unable to detect evidence of divergent selection particular to one or a few study sites. Thus, we tested for divergent selection between elevations within each site through classical coalescent (FDIST) and Bayesian approaches implemented in Arlequin and BAYESCAN respectively, plus a simplified (single-site) version of the Bayesian model introduced in this study (hereafter ‘SBM’).

The coalescent FDIST approach [26], implemented in Arlequin [78], was run under 20000 iterations assuming an island model. Bayescan v.2.1 [28] was run with a burn-in of 50000 iterations, a sample size of 5000, a thinning interval of 10 (resulting in a total of 100000 iterations), and a prior odd of 10000 [32] indicating that for every locus the neutral model is much more likely than the model with selection (*i*.*e*. 10000 neutral loci for every one under selection).

Loci displaying a significant effect of the *α*-component under a *FDR* threshold of 0.1 were retained. Lastly, SBM (a simplified, single-site, Bayesian model excluding genome-wide effects of differentiation among clusters and sub-clusters) was also applied to each site using 10000 iterations with a burn-in period of 5000 iterations.

Within-site outlier-detection tests (FDIST, BAYESCAN and SBM) were applied to both *A*. *alba* and *A*. *cephalonica* study sites. The power of within-site Bayesian methods (BAYESCAN and SBM) was also assessed through simulated data composed of two populations of N = 1600 diploid individuals each (totaling N = 3200 diploid individuals). Monomorphic loci within each site were discarded before within-site analyses.

### 8. Within-site Genotype-Environment Association tests (GEAs)

We assessed whether the genotypic frequencies were structured between elevations within each study site through genotype-environment association tests (see the methodology flowchart in S2 Fig).

For each study site:

1. An empirical distribution of the fixation index (*F*_*IS*_) across loci in the entire population (low and high elevations confounded) was drawn, with:
  $$F_{IS}=1-\frac{H_{obs}}{H_{\text{exp}}}=1-\frac{H_{obs}}{2pq}$$
2. *n1* = 100 *F*_*IS*_*-values* were sorted in the empirical distribution of *F*_*IS*_, and *n1* populations of *N* = 1000 diploids individuals were simulated under Hardy-Weinberg equilibrium corrected by the different *F*_*IS*_*-values*.
3. *n2* = 100 sub-populations pairs of size 500 diploid individuals (corresponding to each elevation in our real dataset) were randomly sorted from the simulated populations of *N* = 1000 individuals.
4. The distribution of expected genotypic frequencies across the *n1*×*n2* = 10000 iterations was then empirically drawn and corresponds to the expected distribution of genotypic frequencies at each elevation of each study site under the hypothesis of no structuring.
5. The cumulative probability of that distribution was drawn, and a p-value was attributed to observed genotypic frequencies at the different loci in the different sub-populations (elevations). It expresses the probability of a given genotype to be more or less present than expected. A negative association means that the genotype is less abundant at this elevation than expected under the hypothesis of balanced genotype frequency between elevations, while a positive association means that the genotype is more abundant at this elevation than expected under the hypothesis of balanced genotype frequency between elevations.

The test was applied to each study site separately. Monomorphic loci were discarded before analyses. The test is written in R and provided in appendix (S2 Method).

## Results

### 1. Genetic structure

STRUCTURE revealed a clear spatial structuring up to *K* = 4, with the highest *ΔK* at *K* = 2 followed by a secondary peak at *K* = 4 (Fig 1 and S3 Fig), suggesting that the most likely number of genetic clusters is 2: one group corresponding to western Europe (French sites) and one group corresponding to central-eastern Europe (Italian, Bulgarian and Romanian sites). The genetic structure remained concordant with the geographical distribution of the studied sites until *K* = 4, at which a secondary *ΔK* peak was detected, suggesting complex hierarchical structuring. At *K* = 4, the clusters detected at *K* = 2 were both split into two sub-clusters: the western Europe cluster into two sub-clusters corresponding to western (Pyrenees) and eastern (Alps) France, and the central-eastern Europe cluster into Italy and Balkan (Romania and Bulgaria) sub-clusters. *K* = 2 and *K* = 4 were thus retained for defining the assignment of each population to a given cluster and sub-cluster in the following hierarchical Bayesian approach (HBM), as described in the Materials and Methods.

### 2. Multi-site outlier detection under the HBM

#### 2.1. Results using simulated data

Running HBM on simulated data revealed high power to detect divergent selection in the replicated population pairs under a hierarchical island model compared to within-site approaches. Table 2 shows a summary of the empirical assessment of *FDR* and *FNR* under the different selection scenarios at 5% and 1% p-value thresholds (see also S4–S7 Figs). *FDR* was always very low, even in the case of a combination of homogenizing and divergent selection and whatever the selection strength (*s*). No outliers were detected at 1% threshold when no selection was applied in spite of strong complex neutral genetic structure among populations, except for one false positive detected at a selection strength (*s*) of 0.5. *FNR* was low under scenarios of strong homogenizing and divergent selection (*FNR* = 0%), but was high under weak to moderate selection, even more when the number of loci was high and the selection strength was variable among loci: at a 1% threshold, the model missed all outliers when a selection strength of 0.05 was applied, and *FNR* varied between 0% and 8% at a selection strength of 0.075 depending on the number of loci under selection. While a 1% threshold may lack true-positives, a 5% threshold may produce false-positives. Thus, it is more conservative to retain only outliers detected at 1% threshold, as it is less damageable to miss outliers than to produce false-positives. For this reason, a threshold of 1% was retained.

**Table 2:** Power of the Bayesian approaches (HBM, SBM and BAYESCAN) to detect outliers for selection from simulated data. False-discovery rates (*FDR*) and false non-discovery rates (*FNR*) were empirically assessed under different scenarios of divergent and/or homogenizing selection, by varying the proportion of selected loci, and selection strength.

				Multi-site analysis (Hierarchical model HBM)						Within-site analysis					
				5% threshold			1% threshold			SBM (threshold 1%)			BAYESCAN (PO = 10000)		
Selection type	Number of selected loci (out of 100)	Selection strength (s)		N detected	FDR (%)	FNR (%)	N detected	FDR (%)	FNR (%)	N detected	FDR (%)	FNR (%)	N detected	FDR (%)	FNR (%)
No selection	0	NA	NA	1	100		0	NA		0	NA		0	0	
Divergent	1	weak	0.05	1	0	0	0	NA	1.00	0	NA	1.00	0	0	1.00
Divergent	5	weak	0.05	1	0	4.04	0	NA	5.00	1	0	4.04	0	0	5.00
Divergent	10	weak	0.05	2	0	8.16	0	NA	10.00	1	0	9.09	0	0	10.00
Divergent	1	moderate	0.075	1	0	0	1	0	0	2	50.00	0	2	50.00	0
Divergent	5	moderate	0.075	5	0	0	3	0	2.06	3	0	2.06	5	0	0
Divergent	10	moderate	0.075	6	0	4.26	2	0	8.16	0	NA	10.00	0	0	10.00
Divergent	1	strong	0.1	1	0	0	1	0	0	1	0	0	1	0	0
Divergent	5	strong	0.1	5	0	0	5	0	0	4	0	1.04	5	0	0
Divergent	10	strong	0.1	10	0	0	3	0	7.22	1	0	9.09	0	0	10.00
Divergent	1	very strong	0.5	2	50.00	0	2	50.00	0	1	0	0	1	0	0
Divergent	5	very strong	0.5	5	0	0	5	0	0	5	0	0	5	0	0
Divergent	10	very strong	0.5	10	0	0	10	0	0	10	0	0	10	0	0
Divergent	1	very strong	0.99	2	50.00	0	1	0	0	1	0	0	1	0	0
Divergent	5	very strong	0.99	5	0	0	5	0	0	5	0	0	5	0	0
Divergent	10	very strong	0.99	10	0	0	10	0	0	10	0	0	11	9.09	0
Divergent	10	variable moderate	0.06–0.15	8	0	2.17	4	0	6.25	1	0	9.09	0	0	10.00
Divergent	10	variable strong	0.16–0.25	11	9.09	0	6	0	4.26	8	0	2.17	11	9.09	0
Divergent	10	variable moderate to strong	0.06–0.24	10	0	0	10	0	0	4	0	6.25	0	0	10.00
Homogenizing	5	strong	0.1	5	0	0	5	0	0	0	NA	5.00	0	0	5.00
Homogenizing / Divergent	2/2	strong	0.1	2/2	0	0	2/2	0	0	2/0	0	2.00	0/2	0	2.00

#### 2.2. Results obtained using *A*. *alba* data

The distributions of inferred *G*_*ST*_ between populations are displayed in S8 and S9 Figs. As expected, the mean *G*_*ST*_ decreased across levels of genetic divergence: the differentiation between populations belonging to different clusters and sub-clusters (*G*_*ST*_ = 0.063, S8A and S8B Fig) was slightly higher than the differentiation between populations belonging to the same cluster but different sub-clusters (*G*_*ST*_ = 0.056 S8C and S8D Fig), which was subsequently higher than the differentiation between populations belonging to the same cluster and the same sub-cluster but different elevations (*G*_*ST*_ = 0.013, S8E Fig). This resulted in decreasing genome-wide parameters inferred by HBM from broad to local scales: *μ*_*Cluste*r_ = 1.3 (with 95%CI = [1.28;1.35]) > *μ*_*SubCluster*_ = 1.14 [1.1;1.19] > *μ*_*Elev*_ = -0.03 [-0.06;-0.001]. The extent of differentiation between elevations within sub-clusters was quite low compared to the extent of differentiation among populations belonging to different clusters (*G*_*ST*_ = 0.063) and sub-clusters (*G*_*ST*_ = 0.056): the *G*_*ST*_ varied between 0.003 in site 9 (Romania) and 0.015 in site 4 (Issole, France) (S9 Fig).

**Fig 2:**
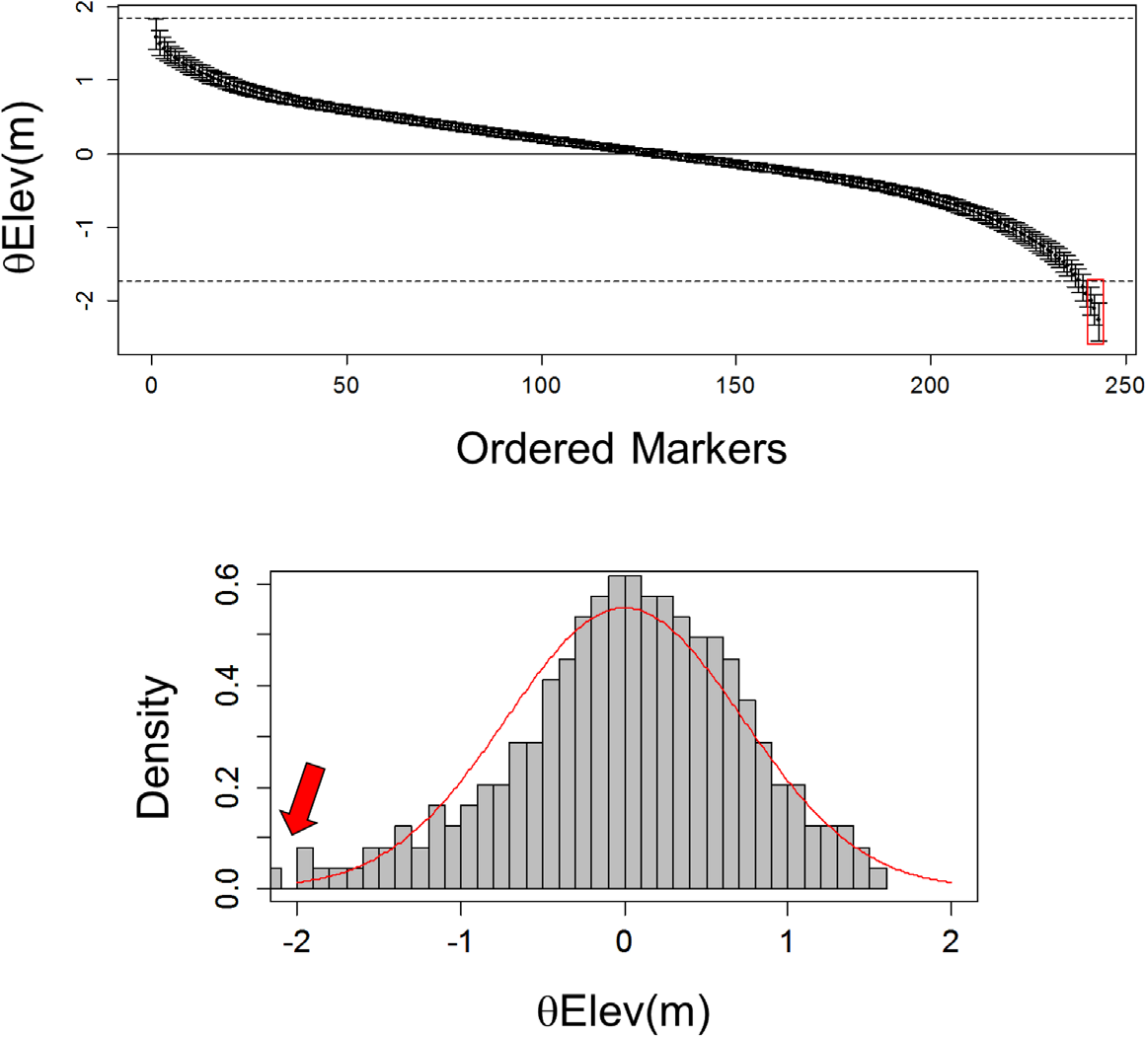
Hierarchical (multi-site) outlier detection. Result of HBM (hierarchical Bayesian approach) on the complete *A*. *alba* dataset. *θ*_*Elev(m)*_ are locus-specific effects on genetic differentiation among populations belonging to different elevations. On the left, the estimated values of *θ*_*Elev(m)*_ with their 95% posterior credible intervals. The markers are sorted by decreasing values of *θ*_*Elev(m)*_ and the dotted lines represent the inter-quantile limits [Q_1_-1.5(Q_3_-Q_1_); Q_3_+1.5(Q_3_-Q_1_)]. On the right, the distribution of *θ*_*Elev(m)*_ and the fitted normal distribution. The arrow indicates the two loci detected below the neutral background in the complete dataset under a 1% probability threshold.

HBM did not detect outliers above the neutral background suggesting no tendency of divergent selection between high and low elevations replicated in the different sites. Two SNPs were detected below the neutral background: SNP 26 and SNP 111 (Fig 2 and S1 Table). However, these two outliers were caused by rare variants rather than by homogenizing selection. SNP 26 was monomorphic except in sites 2 and 5 (Ventoux and Vesubie, East France): the minor allele was brought by three and one heterozygotes at low elevation in each site, respectively. SNP 111 was monomorphic except in site 5 at low elevation and was represented by one homozygote. These two SNPs were removed from within-site outlier detection tests in sites in which they were monomorphic.

### 3. Within-site outlier detection

#### 3.1. Results using simulated data

SBM was applied to simulated data corresponding to individual study sites, and was less powerful than HBM as it failed to detect true positives in many cases: under selection strengths of 0.1 and below, as well as under variable selection strength among loci (Table 2).

The detection power of SBM (with 1% threshold) and BAYESCAN (with prior odd 10000) was quite comparable under scenarios of uniform selection among loci, with minor differences only. However, BAYESCAN was less powerful than SBM under scenarios of variable selection among loci, as it produced more false-positives and more false-negatives.

The hierarchical approach implemented in HBM (under 1% threshold) was generally more powerful than within-site analyses, as the *FDR* was equal to 0% in all cases except one (one loci under very strong selection of strength *s* = 0.5). The *FNR* of HBM was also lower than the *FNR* of intra-site approaches, except in one scenario (10 loci under variable selection among loci, with selection strength ranging between *s* = 0.16 and *s* = 0.25) at which HBM detected only 6 outliers (*i*.*e*. four false-negatives) while SBM detected 8 outliers (*i*.*e*. two false-negatives) and BAYESCAN detected 11 outliers (*i*.*e*. one false-positive).

#### 3.2. Results obtained using *Abies* data

**Fig 3:**
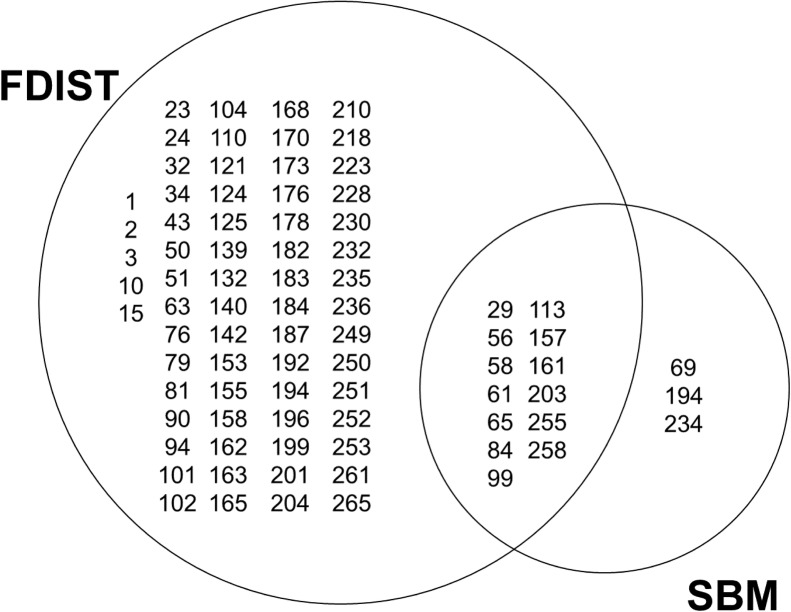
Within-site outlier detection. Results of FDIST and SBM within each *A*. *alba* and *A*. *cephalonica* study sites. It is noteworthy that some outliers were detected in two study sites.

A summary of the outliers detected using the different within-site approaches is provided in Fig 3, Table 3 and S1 Table.

**Table 3:** Consistent outliers detected twice above the neutral background (by two different approaches). The first column describes the SNP number, the second column the SNP ID. The third column describes the study site in which the outliers were detected and the method used: FDIST (within-site coalescent method) and SBM under a 1% threshold (within-site Bayesian method). Study sites IDs are described in Table 1. The complete list of outliers detected, all methods confounded, is provided in S1 Table.

SNP N°	SNP ID	Study site ID ^(method)^
29	contig02088.183	Site 1 ^(FDIST),(SBM)^
58	contig03942.73	Site 9 ^(FDIST),(SBM)^
61	contig04538.344	Site 6 ^(FDIST),(SBM)^
84	contig06968.51	Site 6 ^(FDIST),(SBM)^
113	contig11291.4439	Site 2 ^(FDIST),(SBM)^ / Site 7 ^(FDIST)^
157	contig16125.157	Site 5 ^(FDIST),(SBM)^
161	contig16332.419	Site 1 ^(FDIST)^ / Site 10 ^(FDIST),(SBM)^
203	contig20694.1090	Site 7 ^(FDIST),(SBM)^
255	contig09373.367	Site 9 ^(FDIST),(SBM)^
65	contig05004.249	Site 1 ^(FDIST),(SBM)^
99	contig08649.617	Site 5 ^(FDIST),(SBM)^
258	contig15452.813	Site 2 ^(FDIST),(SBM)^

The coalescent approach (FDIST) detected a total of 97 outliers, all sites confounded: 64 outliers were below the neutral background and 33 outliers were above the neutral background (S1 Table). The number of outliers in each study site detected by FDIST varied between five in the site 7 (Italy ‘Colle dell’Abete’) and twenty-two in site 9 (Romania). In site 8 (Bulgaria), the differentiation between elevations was too low to infer meaningful migration rates and to identify outliers through FDIST.

SBM detected a total of 16 outliers (S10 Fig and S1 Table) located in different transcripts: three in site 1 (SNPs 29, 65 and 194), four in site 2 (SNPs 56, 69, 113, and 258), three in site 5 (SNPs 99, 157 and 234), two in site 6 (SNPs 61 and 84), one in site 7 (SNP 203), two in site 9 (SNP 58 and 255), one in site 10 (SNP 161). In particular, SBM validated 12 outliers above the neutral background detected by FDIST (SNPs 29, 58, 61, 65, 84, 99, 113, 157, 161, 203, 255 and 258, Fig 3 and S10 Fig) and detected three additional outliers below the neutral background not detected by FDIST (SNPs 69, 194 and 234). Additionally, SNP 56 was detected below the neutral background by both FDIST and SBM but in different sites (1 and 2 respectively).

BAYESCAN detected no outliers with a prior odd of 10000.

### 4. Within-site Genotype-Environment Association tests (GEAs)

GEAs were conducted in order to address the question of altitudinal adaptation at the genotype level. GEAs aimed at testing whether a genotype was more or less abundant at a given elevation than expected under the hypothesis of balanced genotypic frequencies between elevations. Among the 13374 associations tested (all sites, elevations, SNPs and genotypes confounded), 10306 (77.06%) were non-significant, while 3068 (22.94%) were significant, suggesting either a positive or a negative association of a given genotype (homozygote or heterozygote) at a given (low or high) elevation.

Because a large proportion of outliers under divergent selection previously detected are expected to be false-positives, we further focused on the 12 consistent outliers for divergent selection detected twice by the two independent approaches FDIST and SBM respectively (Table 3). For all of them, significant GEAs were observed in the study sites where they were detected, with contrasted patterns between low and high elevations (S2 Table). For example, at SNP 157, homozygotes (GG) were more abundant at low elevation than expected under the hypothesis of balanced genotypic frequencies between elevations (resulting in a positive association) in the study site 5 (where this SNP was detected as outlier for divergent selection). Homozygotes (GG) were also less abundant at high elevation than expected (resulting in a negative association) and heterozygotes (AG) were more abundant at high elevation than expected (resulting in a positive association).

## Discussion

### 1. Power of Bayesian modelling approaches (HBM, SBM and BAYESCAN) to detect homogenizing and divergent selection in replicated population pairs (simulations)

Bayesian approaches are known to be more stringent than methods based on coalescent simulations for identifying *F*_*ST*_-outliers [36, 50]: even if Bayesian approaches may miss some true-positives, they often produce less false-positives than the coalescent approach by Beaumont and Nichols [26]. This observation was confirmed in our study, where significantly more outliers were detected using FDIST than using BAYESCAN or SBM when applied to *A*. *alba* data (S1 Table).

The power of BAYESCAN (with prior odd 10000) and SBM was also explored in more details through simulated data, revealing minor differences in the detection power of the two approaches (Table 2). These differences are probably caused by differences between the two methods, including mathematical formulation, programming, Markov Chain Monte Carlo sampling algorithm, and decision strategy. Especially, BAYESCAN and the Bayesian models developed here (HBM and SBM) use different differentiation indices: BAYESCAN estimates a locus-specific-population *F*_*ST*_ close to Wright’s *F*_*ST*_, while the models introduced here use pairwise Nei’s *G*_*ST*_. Locus-specific *G*_*ST*_ values were globally lower than *F*_*ST*_ values (*μ* = 0.010 *vs* 0.017), but also more variable (*σ* = 0.013 *vs* 0.010) which probably facilitates the inference of locus-specific parameters (S11 Fig). In addition, HBM and SBM partition pairwise *G*_*ST*_ between populations into genome-wide and locus-specific parameters, while BAYESCAN partitions locus-population-specific *F*_*ST*_ coefficients that measure the shared ancestry within each of the subpopulations. Moreover, the models encoded here do not use a composite likelihood to identify outliers and thus do not require locus-specific prior odds. Instead, there is no *a priori* assumption and a p-value is directly attributed to each marker from the inferred normal distribution of locus-specific parameters, leading to faster computation and allowing the detection of outliers below and above the neutral background.

Simulations revealed the high power of the hierarchical approach to detect both homogenizing and divergent selection in replicated population pairs: the false discovery rate was always very low, and outliers detected under a 1% threshold may be reasonably considered as truly under selection (Table 2 and S5–S7 Figs). However, HBM may fail to detect some loci under selection and the false-non-discovery rate may be high when the selection strength is weak (because the extent of differentiation is not strong enough to be outside the neutral background), particularly when many loci are selected and the selection strength is variable among loci (because the distribution of locus-specific parameters is empirically drawn and becomes wider when the number of selected loci increases). Nevertheless, HBM was more powerful than within-site Bayesian approaches (BAYESCAN and SBM) as it detected no (or few) false-positives with a *FNR* lower than intra-site approaches. In particular, it was able to detect replicated patterns of locus-specific divergence caused by moderate to strong selection strength that have not been detected when analyzing the different study sites independently through SBM (Table 2). This reinforces the idea that testing for local adaptation not only requires replicated population pairs at different study sites, but also the use of *ad hoc* models able to take into account replicated sites simultaneously with respect to their genome-wide genetic structure. On the other hand, HBM was designed to detect patterns of local adaptation replicated in the different study sites, but it was intrinsically unable to detect divergent selection particular to one or a few sites. Therefore, such hierarchical approach remains highly sensitive to heterogeneous selection among replicated population pairs, and testing for adaptation within each site remains essential to finely explore the process of adaptation within sites spread across broad geographical scales.

### 2. Altitudinal adaptation across the southern distribution range of *Abies alba*

Altitudinal gradients have attracted much attention because they display strong ecological variations (*e*.*g*. temperature, solar irradiance and precipitation) over short geographical scales [10, 37, 79, 80], and many studies have provided evidence of local adaptation to elevation in a variety of plant species at both phenotypic [81–83] and molecular levels [18, 19, 61, 84–88]. This study addresses the question of altitudinal adaptation across large spatial scales, from Western to Eastern Europe, using a dataset composed of 273 expressed SNPs located in candidate genes.

The analysis of neutral genetic variation showed a clear hierarchical genetic structure into clusters (*K* = 2) and sub-clusters (*K* = 4), as further confirmed by decreasing estimates of genome-wide parameters across geographical scales (*μ*_*Cluster*_> *μ*_*SubCluster*_> *μ*_*Elev*_). This observation was concordant with a classical ‘isolation-by-distance’ model, in which the extent of neutral genetic differentiation increases with geographic distance because of limitations in gene flow caused by the geographic distance itself.

The hierarchical Bayesian model (HBM) detected only two SNPs below the neutral background (SNPs 26 and 111) but no common trend of divergent selection between elevations in sites spread across the whole southern distribution of silver fir. On the contrary, within-site analyses allowed the detection of many outliers (S1 Table) but most of them are probably false-positives, as the majority was detected in only one study site and by the coalescent (FDIST) approach only, and not validated by any of the Bayesian approaches (SBM or BAYESCAN). These genes may either be neutral or under too weak selection strength to be detected by the Bayesian approaches, thus making the distinction between true and false-positives extremely complicated. Especially, the demographic histories experienced by populations inhabiting the different sites (for example bottleneck or fast expansion) would also affect allele frequency spectra, resulting in the discovery of false-positives or false-negatives by selection tests [27, 89, 90]. Even if Bayesian approaches [25, 28] are known to be less sensitive to neutral processes and less prone to detect false-positives than the coalescent approach [36, 50, 91], they may remain sensitive to demographic processes [32, 36, 50, 55, 56, 92]. However, the confounding effects of demography and selection have not been fully explored yet, and it is impossible to conclude whether the genes showing a departure from neutral expectations in one site and only detected by a single approach are true-positives or not, without knowing if the different populations have experienced strong demographic changes. In addition, the relative measures of divergence used by these methods (*F*_*ST*_, *G*_*ST*_) are sensitive to heterogeneous recombination rates in the genome. For example, centromeric or rearranged regions, where recombination is reduced, are expected to be more differentiated than the rest of the genome [34], and SNPs located in these regions are likely to be false-positives. However, the location of the targeted contigs and SNPs on the chromosomes of the species is unfortunately unknown. At last, the SNPs detected below the neutral background cannot be viewed as being involved in local adaptation caused by elevation and will not be considered thereafter.

Twelve SNPs (Table 3) may nonetheless be reasonably considered under divergent selection in one study site (SNPs 29, 58, 61, 65, 84, 99, 113, 157, 161, 203, 255 and 258), as they were detected above the neutral background by both FDIST and SBM and they were corroborated by contrasted GEAs between elevations at study sites in which they were detected. It is however unclear why these 12 SNPs were not detected by BAYESCAN, and several reasons may be invoked: (i) the selection strength may have been too weak to allow their detection by BAYESCAN (as suggested by the results on simulated data), (ii) the dataset size may be too small to allow a reliable outlier detection, and/or (iii) a prior odd of 10000 may be too stringent to detect outliers in scans of expressed candidate SNPs [88]. In fact, prior odds up to 10000 (that means that each SNP is ten thousand times more likely to be neutral than to be under selection) are commonly used to identify candidate loci in the context of genome wide association studies with millions of SNPs. However, each SNP analyzed through a genome scan of candidate genes is expected to be as much or more likely selected than neutral, and a prior odd of 10000 would probably lack true positives in this context. In addition, local demographic events may also produce false-positives, but the sensitivity of the different methods used (FDIST, BAYESCAN, SBM) has not been explored in this study.

These 12 consistent outliers (out of 273 analyzed) were particular to one study site, and the proportion of consistent outliers within each study site varied between 0% and 0.95% (S3 Table). These estimates may however be biased by the size of the dataset. Indeed, this study focused on a set of 273 SNPs within 177 transcripts, which is far from being representative of the entire genome, and local adaptation may have targeted other genes not examined in this study. Moreover, the SNPs analyzed here are located in expressed candidate genes and therefore this set of SNPs is probably enriched in selected SNPs, which may result in an over-estimation of the true proportion of selected SNPs in the entire genome. Nevertheless, these estimates are comparable to those obtained by other experimental surveys of altitudinal adaptation in *Fagus sylvatica*. In the Mediterranean area, Jump *et al*. [93] detected one outlier locus out of 241 scored (0.4%) in the Mountains of Catalonia in north-eastern Spain, while Csilléry *et al*. [88] detected only three marginally significant outliers out of 546 scored SNPs (0.5%) in the Mont Ventoux study site in south-eastern France. Also, Pluess and Weber [94] detected 13 outlier loci out of 517 scored (2.5%) in the lowland forests of Switzerland. In *Abies alba*, adaptive variations have recurrently been reported for quantitative traits, isozymes, and candidate genes (sequences and SNPs data) in European wild populations [18, 60–63]. In particular, Roschanski *et al*. [62] recently provided evidence of local adaptation to drought and cold tolerance in the French Alps at both molecular and phenotypic levels. They identified 16 outlier out of 267 SNPs (~6%) that showed patterns of divergent selection, and 8 genes containing SNPs at which allelic frequencies were correlated with bioclimatic variables. Among the SNPs they analyzed, one (0.37%) showed evidence of adaptation to altitude. In addition, two consistent SNPs detected here (SNP 65 in the Pyrenees and SNP 157 in the French Alps) were also detected as being involved in adaptation by Roschanski *et al*., providing thus additional evidence of local adaptation in *A*. *alba*.

**Fig 4:**
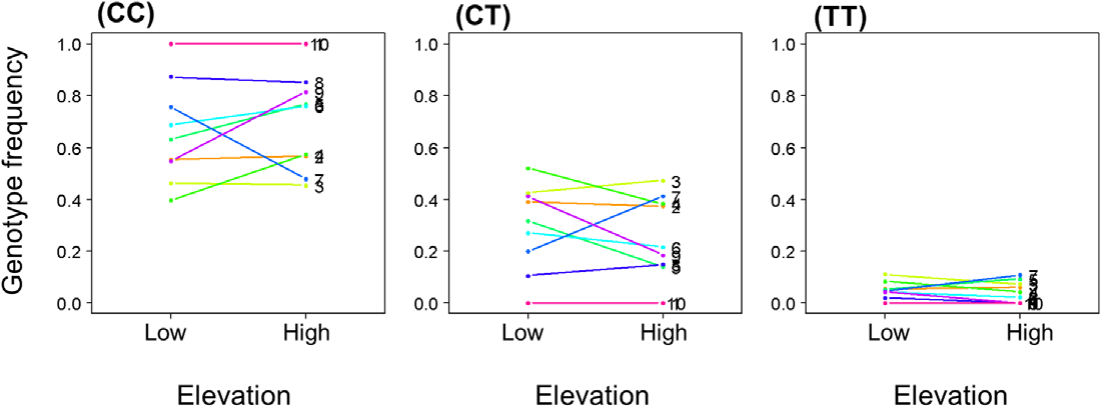
Idiosyncrasy between sites. Observed genotypic frequencies in the different sites at SNP 255 (genotypes CC, CT and TT). SNP 255 was detected by two within-site outlier detection methods (FDIST and SBM) in site 9 (purple line), but in no other site. In addition, significant GEAs were detected in site 9 (S2 Table).

Taken together, the results obtained through hierarchical and within-site approaches revealed only weak evidence of local adaptation to altitude, as idiosyncratic patterns among study sites were detected by the within-site FDIST and SBM approaches only (see for example variations in genotypic frequencies among sites at SNP 255, Fig 4). It is however not surprising to find site-specific evidence of selection not detected by the hierarchical (multi-site) approach, as the process of local adaptation to elevation is probably confounded with the processes of adaptation caused by large-scale factors not explored in this study (*i*.*e*. variations in environmental conditions between clusters, sub-clusters or sites). Indeed, the analyzed study sites may not be exactly ‘true’ replicates of the altitudinal contrasts as the range of ecological conditions might vary among them. In particular, the sampled populations occur at different elevations (Table 1), and the ecological conditions they experience may differ in many ways, including temperature, solar irradiance, length of the growing season, rainfall, soil type, water and nutrients availability and/or pathogens. In addition, the populations inhabiting the different study sites belonged to different lineages/gene pools as revealed by the complex biogeographic structure from West to East Europe: these populations may thus have adapted differently because different ancestral gene pools were exposed to selection. It is thus not surprising that different loci would have been under selection in the different study sites.

The 12 consistent outliers for divergent selection detected twice by within-site approaches and validated by significant GEAs were the strongest possible targets of natural selection, and are thus good candidates for exploring in detail the molecular bases of altitudinal adaptation in *Abies*. These SNPs are probably independent and not significantly linked to each other, or to other SNPs not analyzed in this study. They are located in different expressed genes (*i*.*e*. contigs, with size varying between 418 and 2340 bp, S4 Table) and we do not expect the presence of other -not genotyped- SNPs in the same transcripts or in other transcripts adjacent to those targeted in this study. Moreover, linkage disequilibrium is supposed to decay fast within genes in natural settings (within 100 bp), as suggested by Heuertz *et al*. [95] and Pavy *et al*. [96] in other coniferous species. For example, SNPs 157 and 158 were located in the same contig (428 bp apart) but only one of them (SNP 157) was detected by SBM and considered as a consistent outlier for selection. It confirms that linkage disequilibrium decreases sufficiently fast to prevent hitchhiking between close SNPs, and it proves the robustness of the Bayesian method SBM to potential linkage between a selected SNP and neutral SNPs located in the same or in adjacent contigs.

In this respect, functional annotation may help to identify the metabolic pathways as well as the biological process involved in local adaptation (S4 Table). Especially, it needs to be stressed that six consistent outliers are located in transcripts involved in primary metabolic processes (SNPs 29, 58, 84, 113, 157 and 161) and three in a gene involved in nitrogen compound metabolic process (SNPs 65, 99 and 258). Primary metabolic processes -among which photosynthesis, carbon assimilation and sugar metabolism- are crucial for plants survival and development, and our results suggest that genes involved in primary metabolism pathways would be targeted by natural selection in European fir species. In particular, solar irradiance is a major constraint for plant development, and the processes related to photosynthesis and carbon assimilation have repeatedly been identified as being targets of natural selection in plant populations [10, 97–101]. In addition, the primary products of photosynthesis and the activity of enzymes involved in sugar metabolism may vary with altitude as demonstrated by Kumar *et al*. [102] in grass species. Our results open the question of how metabolic variation in photosynthetic metabolism, sugar biosynthesis and their regulatory pathways may be genetically driven and structured by natural selection along altitudinal gradients. However, fleshing out our understanding on local adaptation to altitude would require a more detailed investigation of the adaptation process at the phenotypic level through quantitative genetic approaches (such as reciprocal transplants), and a fine exploration of the genetic architecture behind metabolic, physiological and morphological variations in wild populations (*via* association genetics and QTL analyses).

## Supporting Information

S1 Fig Hierarchical design. Hierarchical design used for the simulated data and *A*. *alba* datasets. For simulations, migration rates ‘m1’, ‘m2’ and ‘m3’ refers to the migration rate between clusters (*m*_*inter-clusters*_), between sub-clusters within clusters (*m*_*inter-subclusters*_), and within sub-clusters respectively (*m*_*inter-populations*_).
(TIF)

S2 Fig Within-site genotypes-environment associations (GEAs). Methodology flowchart.
(TIF)

S3 Fig Genetic structure. Evanno’s ΔK posterior to STRUCTURE analysis.
(TIF)

S4 Fig Results of HBM on simulated data under a scenario of no selection. The points show the inferred *θ*_*Elev(m)*_ with their 95% credible intervals. The dotted lines represent the inter-quantile limits [Q_1_-1.5(Q_3_-Q_1_); Q_3_+1.5(Q_3_-Q_1_)].
(TIF)

S5 Fig Results of HBM on simulated data under scenarios of divergent selection (uniform among loci). One to 10 loci (out of 100) were submitted to divergent selection between elevations, with a uniform selection strength among loci (*s*) varying between 0.05 and 0.99. The plots show inferred *θ*_*Elev(m)*_ with their 95% credible intervals in the most extreme cases. The dotted lines represent the inter-quantile limits [Q_1_-1.5(Q_3_-Q_1_); Q_3_+1.5(Q_3_-Q_1_)]. The absolute number of outliers detected in each case is shown at the top-right of the different plots: (a) 1% threshold, (b) 5% thresholds.
(TIF)

S6 Fig Results of HBM on simulated data under scenarios of divergent selection (variable among loci). Ten loci (out of 100) were submitted to divergent selection between elevations, with a variable selection strength among loci: weak selection (*s* = [0.05,0.15], left panel), intermediate selection (*s* = [0.15,0.25], middle panel), and wide selection (*s* = [0.05,0.25], right panel). The plots show the inferred *θ*_*Elev(m)*_ with their 95% credible intervals. The dotted lines represent the inter-quantile limits [Q_1_-1.5(Q_3_-Q_1_); Q_3_+1.5(Q_3_-Q_1_)]. The absolute number of outliers detected in each case under 1% (a) and 5% (b) thresholds is shown at the top-right of the different plots.
(TIF)

S7 Fig Results of HBM on simulated data under scenarios of homogenizing selection between elevations (left panel) and of a combination of homogenizing and divergent selection between elevations (right panel). In the left panel, 5 loci (out of 100) were submitted to homogenizing selection (*s* = 0.1, uniform among loci). In the right panel, 4 loci (out of 100) were submitted to selection: 2 under homogenizing selection (*s* = 0.1, uniform among loci), and 2 under divergent selection (*s* = 0.1, uniform among loci). The plots show the inferred *θ*_*Elev(m)*_ with their 95% credible intervals. The dotted lines represent the inter-quantile limits [Q_1_-1.5(Q_3_-Q_1_); Q_3_+1.5(Q_3_-Q_1_)]. The absolute number of outliers detected in each case under 1% (a) and 5% (b) thresholds is shown at the top-right of the different plots.
(TIF)

S8 Fig Inferred *G*_*ST*_ in *A*. *alba*. Distributions of locus-specific *G*_*ST*_ among pairs of *A*. *alba* populations (*site×elevation*) inferred using the hierarchical approach (HBM). Population pairs were classified depending on their membership to the same cluster (*K* = 2), sub-cluster (*K* = 4), and elevation according to the hierarchical approach, cases (a) to (f). The table included below shows the mean *G*_*ST*_ for each case (a) to (f) and details how *G*_*ST*_ values are partitioned into genome-wide and locus-specific effects by HBM. Notice that the parameters *μ*_*Clus*_, *μ*_*SubClus*_ and/or *μ*_*Elev*_ are not applied when the populations (*i*,*j*) belong to the same cluster (*k*_*Clus(i*,*j)*_ = 0), to the same sub-cluster (*k*_*SubClus(i*,*j)*_ = 0), and/or to the same elevation (*k*_*Elev(i*,*j)*_ = 0).
(TIF)

S9 Fig Inferred *G*_*ST*_ in *A*. *alba*. Distribution of locus-specific *G*_*ST*_ between elevations within each study site inferred through classical ‘within-site’ approach (SBM). The values above the plot show the mean differentiation among all markers in each site. Sites ID are described in Table 1.
(TIF)

S10 Fig Within-site outlier detection in *A*. *alba*. Results of SBM within each *A*. *alba* and *A*. *cephalonica* site. The arrows indicate the detected outliers for homogenizing (left-tail) and divergent (right-tail) selection under 1% threshold. Sites IDs are described in Table1.
(TIF)

S11 Fig Within-site genetic differentiation in *A*. *alba*. Comparison of locus-specific differentiation indices estimated within the different sites: *F*_*ST*_ is estimated using BAYESCAN and Nei’s *G*_*ST*_ is estimated using SBM. Different symbols were used for the different sites.
(TIF)

S1 Method Populations simulation. (PDF)

S2 Method Within-site genotypes-environment associations (GEAs). R script and functions.
(PDF)

S1 Table Outliers detection in *A*. *alba* and *A*. *cephalonica* using different approaches (hierarchical and within-site approaches). Outliers are sorted by their position relative to the neutral background: I. Outliers detected above the neutral background; II. Outliers detected above the neutral background in one site and below the neutral background in another site; III. Outliers detected below the neutral background. The first column describes the SNP number, the second column the SNP ID. The third column describes the study site in which the outliers were detected and the method used: ^(a)^ HBM under a 1% threshold (hierarchical multi-site Bayesian method), ^(b)^ FDIST (within-site coalescent method), ^(c)^ BAYESCAN (within-site Bayesian method), ^(d)^ SBM under a 1% threshold (within-site Bayesian method). Study sites IDs are described in Table 1. The 12 outliers detected twice above the neutral background (by two different approaches) are shaded in grey.
(PDF)

S2 Table Results of GEAs for the 12 consistent candidate SNPs for divergent selection between elevations. The first column describes the SNP number, the second column the SNP ID, the third column describes the study site in which the SNP was detected as outlier for divergent selection (study sites IDs are described in Table 1) and the fourth column describes the alleles genotyped. The columns 5 to 10 describes the results of GEAs at low (columns 5 to 7) and high elevations (columns 8 to 10) for each genotype (homozygotes and heterozygote): ‘Homozygote 1’ is the first homozygote and ‘Homozygote 2’ is the second homozygote (for *e*.*g*.: for two alleles (C) and (T), (CC) is the homozygote 1 and (TT) the homozygote 2); ‘+’ indicates a positive association, ‘-’ indicates a negative association, ‘ns’ a non-significant association, and ‘NA’ a missing value (because the SNP is monomorphic in the study site.
(PDF)

S3 Table Proportion of consistent outliers detected in each study site. (PDF)

S4 Table BlastX and functional annotation of the transcripts containing the 12 consistent candidate SNPs for divergent selection between elevations. (PDF)
