# Supplementary figures and images for: Local Adaptation in European Firs Assessed through Extensive Sampling across Altitudinal Gradients in Southern Europe
Source: PLoS One. 2016 Jul 8;11(7):e0158216. doi: 10.1371/journal.pone.0158216 (PMC4938419; doi:10.1371/journal.pone.0158216)

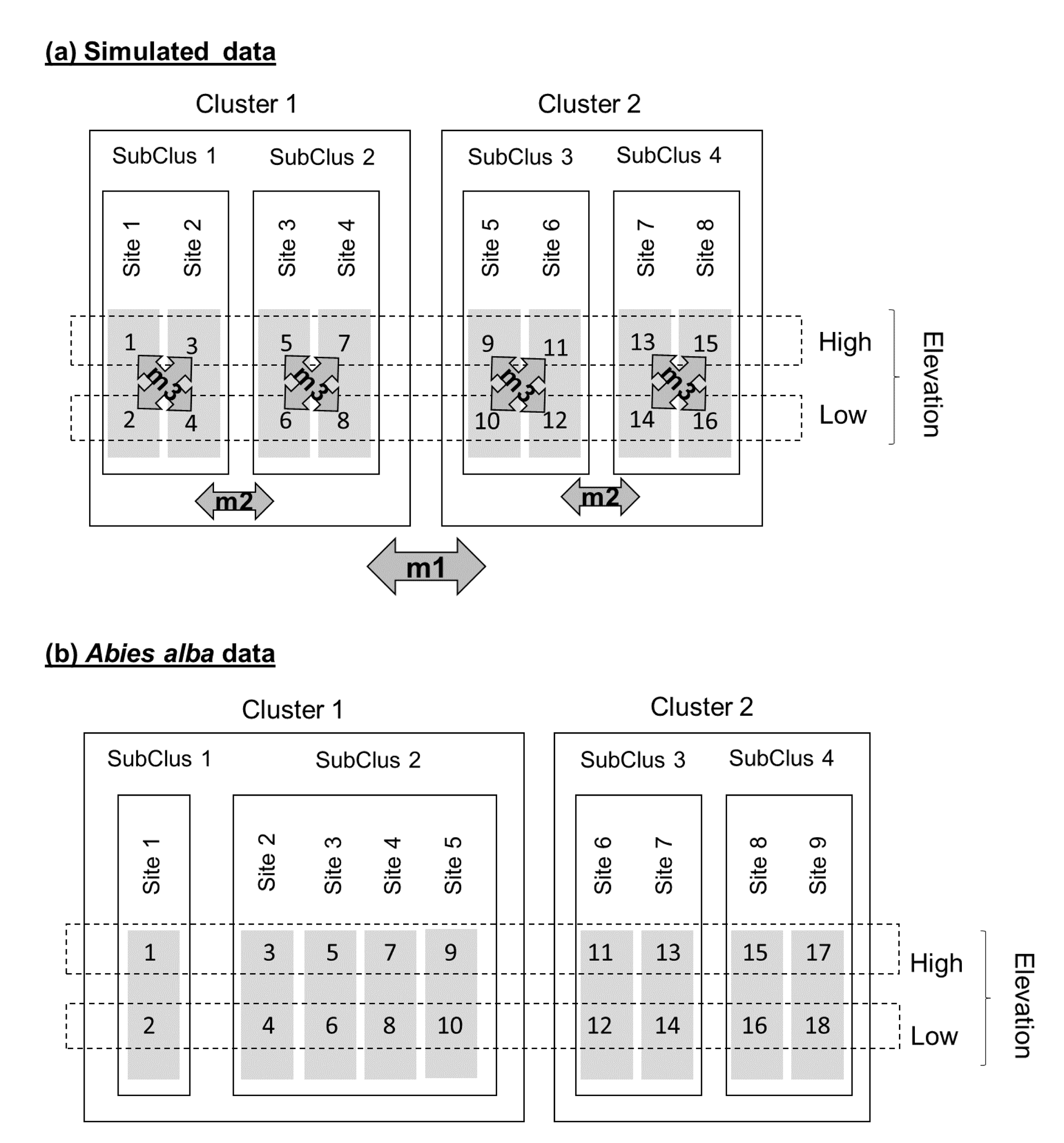

Supplement: S1 Fig — Hierarchical design used for the simulated data and A. alba datasets. For simulations, migration rates ‘m1’, ‘m2’ and ‘m3’ refers to the migration rate between clusters (minter-clusters), between sub-clusters within clusters (minter-subclusters), and within sub-clusters respectively (minter-populations). (TIF) [file pone.0158216.s001.tif]

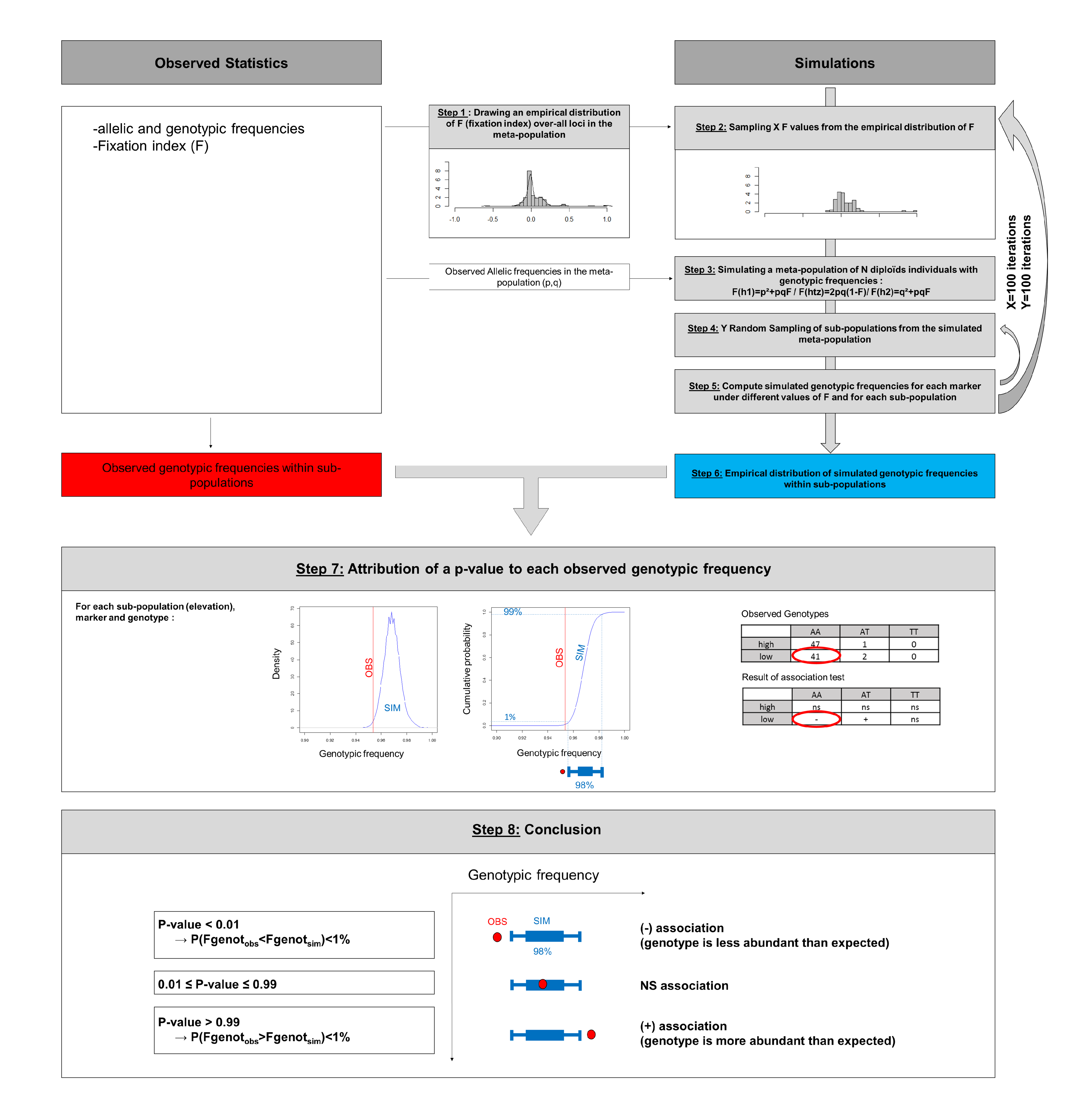

Supplement: S2 Fig — Methodology flowchart. (TIF) [file pone.0158216.s002.tif]

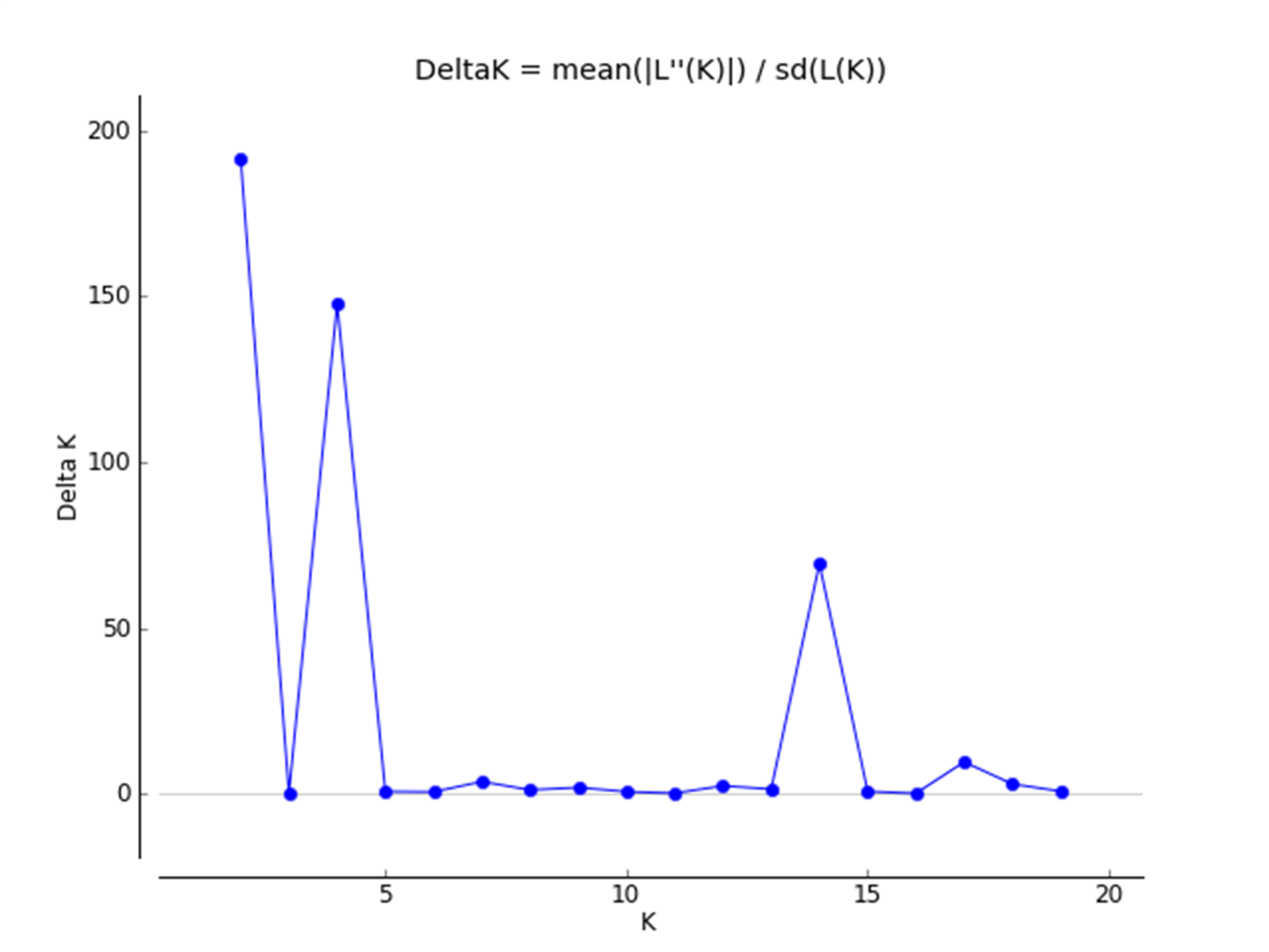

Supplement: S3 Fig — Evanno’s ΔK posterior to STRUCTURE analysis. (TIF) [file pone.0158216.s003.tif]

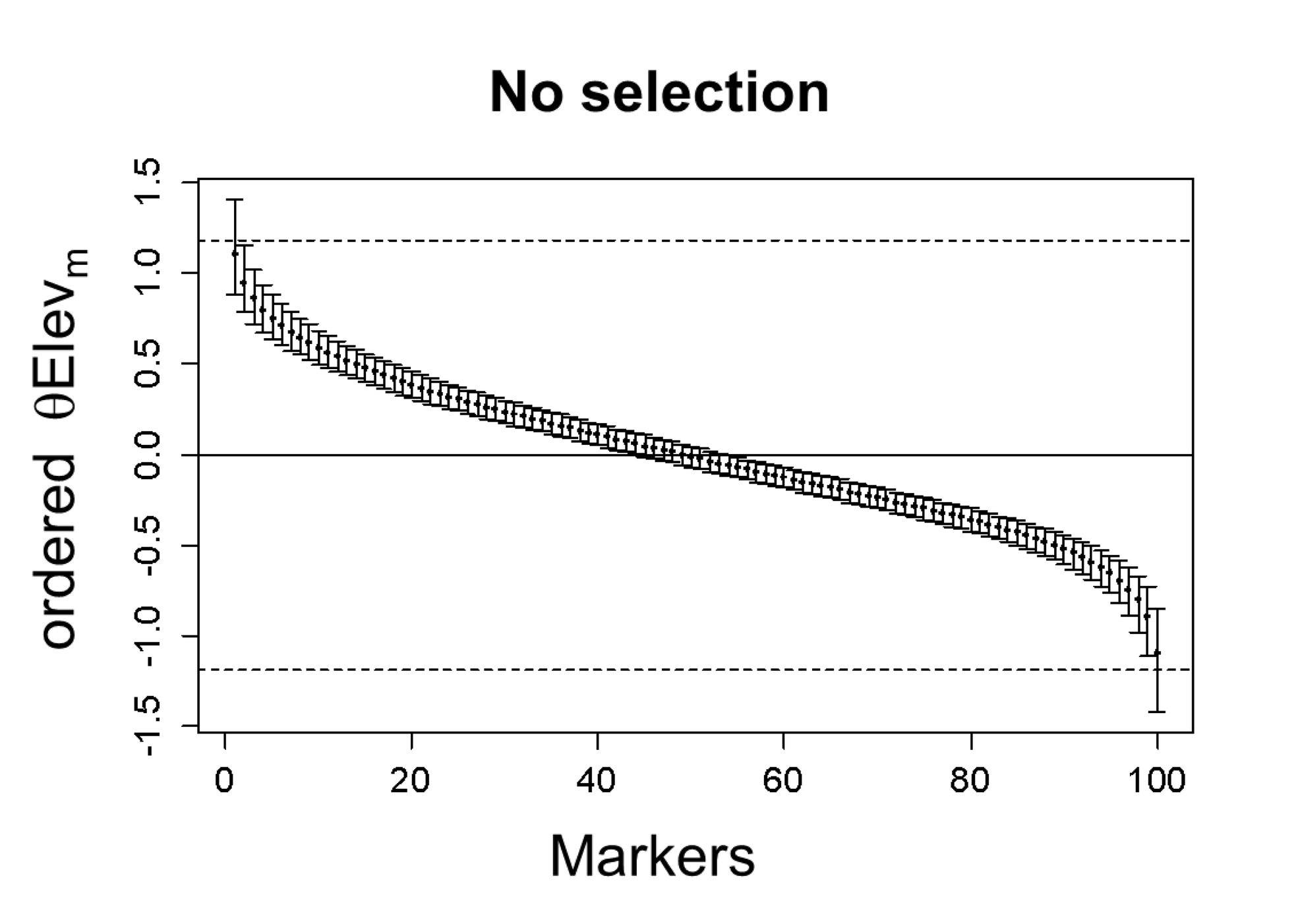

Supplement: S4 Fig — The points show the inferred θElev(m) with their 95% credible intervals. The dotted lines represent the inter-quantile limits [Q1-1.5(Q3-Q1); Q3+1.5(Q3-Q1)]. (TIF) [file pone.0158216.s004.tif]

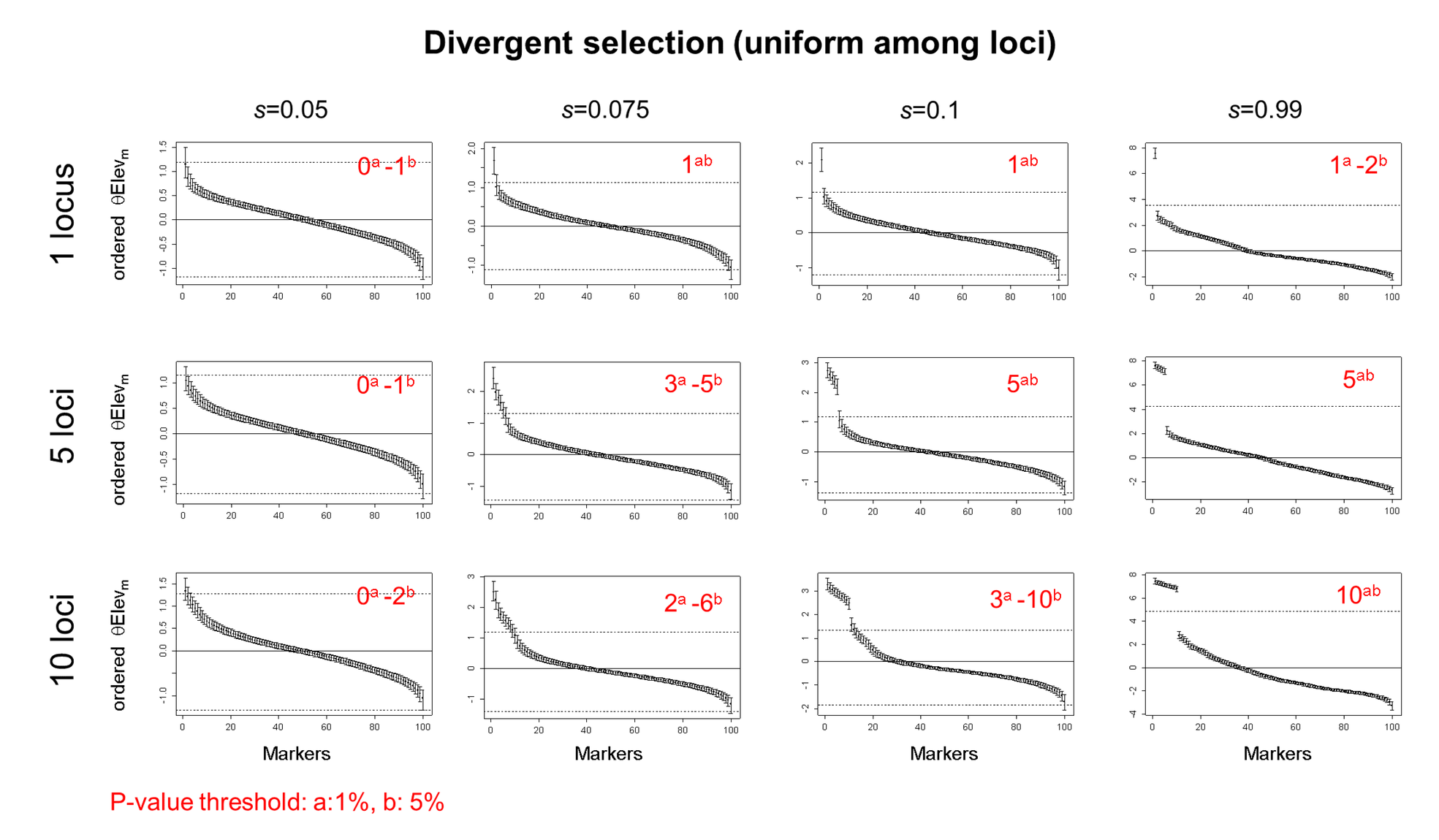

Supplement: S5 Fig — One to 10 loci (out of 100) were submitted to divergent selection between elevations, with a uniform selection strength among loci (s) varying between 0.05 and 0.99. The plots show inferred θElev(m) with their 95% credible intervals in the most extreme cases. The dotted lines represent the inter-quantile limits [Q1-1.5(Q3-Q1); Q3+1.5(Q3-Q1)]. The absolute number of outliers detected in each case is shown at the top-right of the different plots: (a) 1% threshold, (b) 5% thresholds. (TIF) [file pone.0158216.s005.tif]

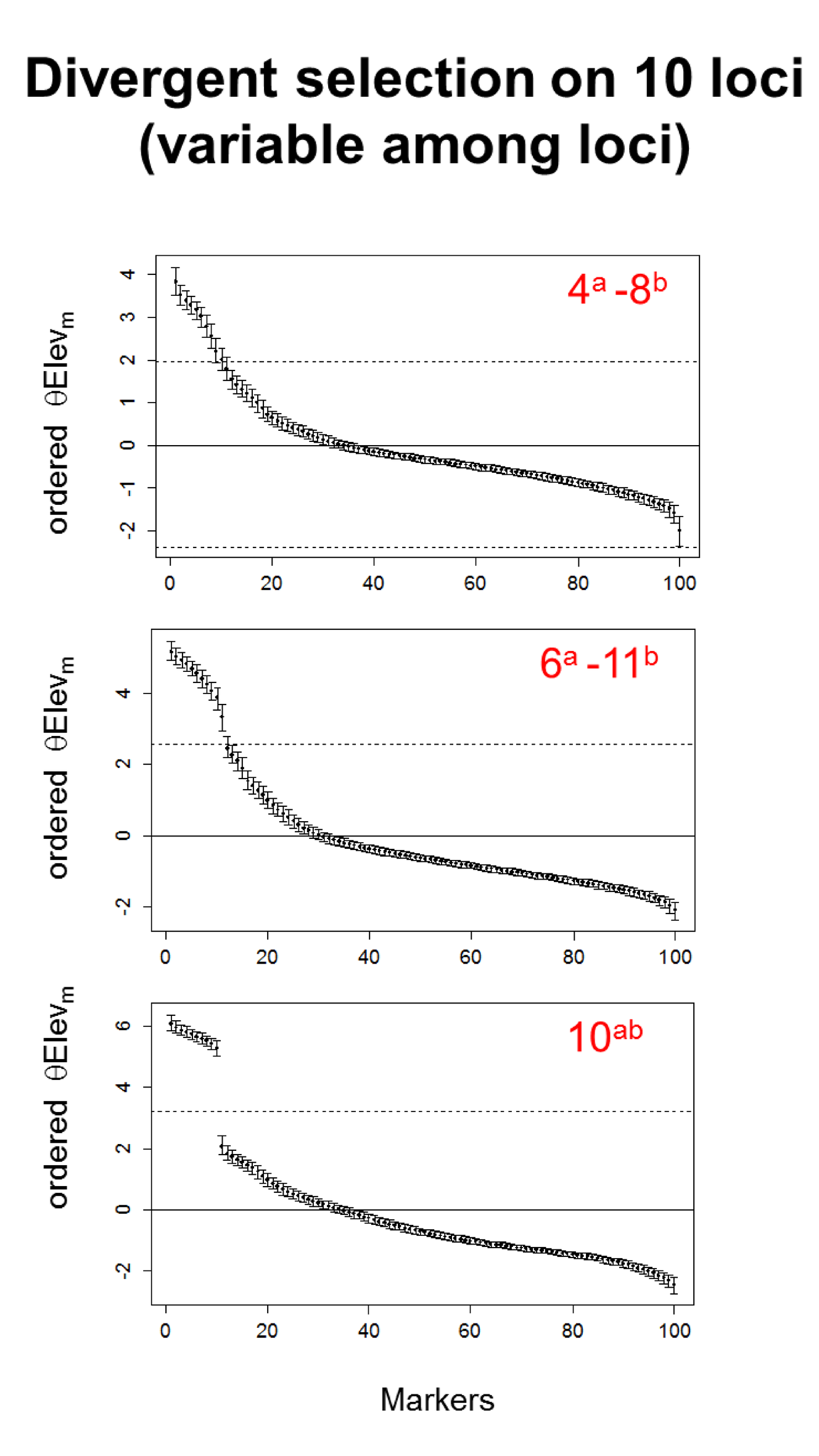

Supplement: S6 Fig — Ten loci (out of 100) were submitted to divergent selection between elevations, with a variable selection strength among loci: weak selection (s = [0.05,0.15], left panel), intermediate selection (s = [0.15,0.25], middle panel), and wide selection (s = [0.05,0.25], right panel). The plots show the inferred θElev(m) with their 95% credible intervals. The dotted lines represent the inter-quantile limits [Q1-1.5(Q3-Q1); Q3+1.5(Q3-Q1)]. The absolute number of outliers detected in each case under 1% (a) and 5% (b) thresholds is shown at the top-right of the different plots. (TIF) [file pone.0158216.s006.tif]

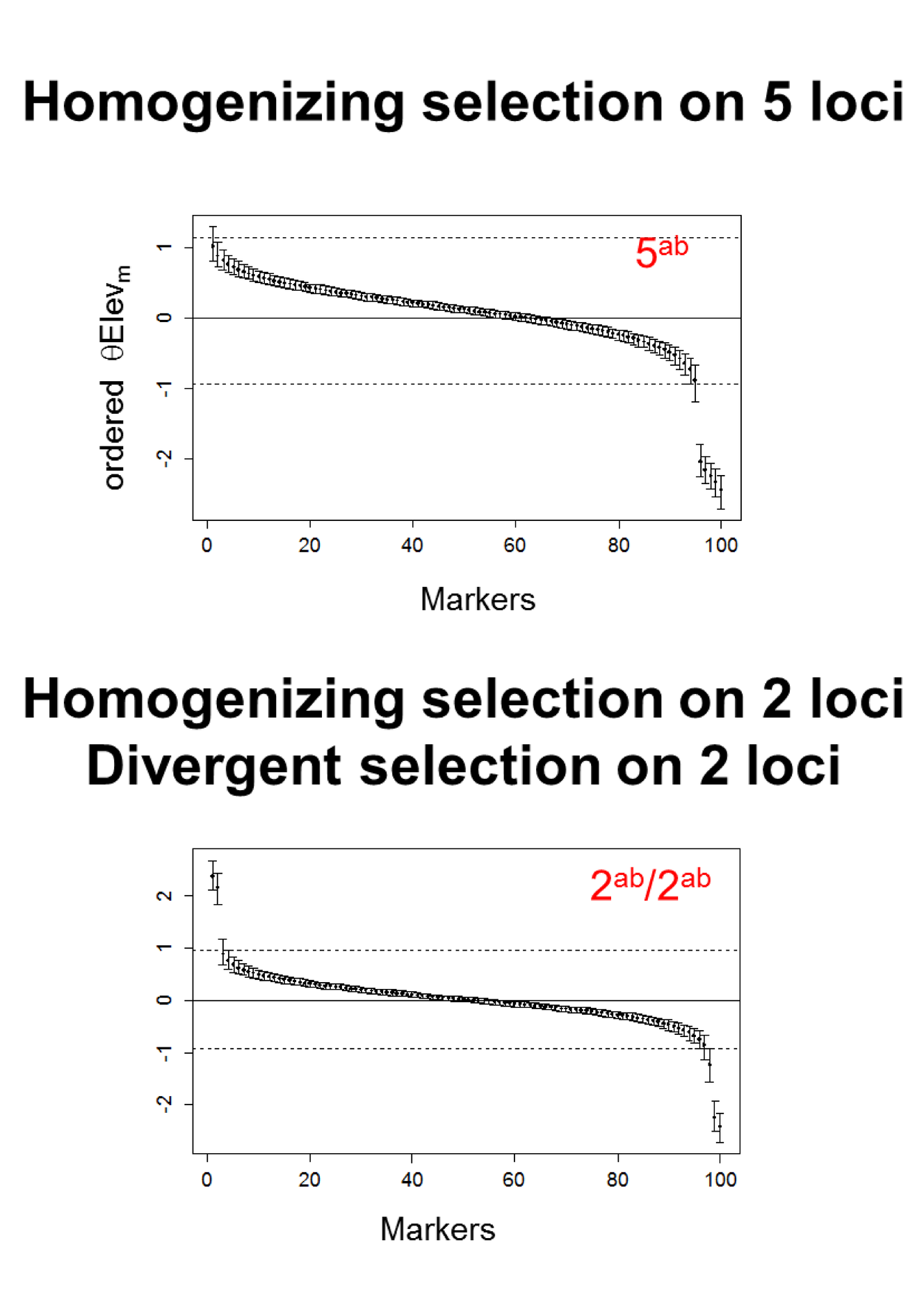

Supplement: S7 Fig — In the left panel, 5 loci (out of 100) were submitted to homogenizing selection (s = 0.1, uniform among loci). In the right panel, 4 loci (out of 100) were submitted to selection: 2 under homogenizing selection (s = 0.1, uniform among loci), and 2 under divergent selection (s = 0.1, uniform among loci). The plots show the inferred θElev(m) with their 95% credible intervals. The dotted lines represent the inter-quantile limits [Q1-1.5(Q3-Q1); Q3+1.5(Q3-Q1)]. The absolute number of outliers detected in each case under 1% (a) and 5% (b) thresholds is shown at the top-right of the different plots. (TIF) [file pone.0158216.s007.tif]

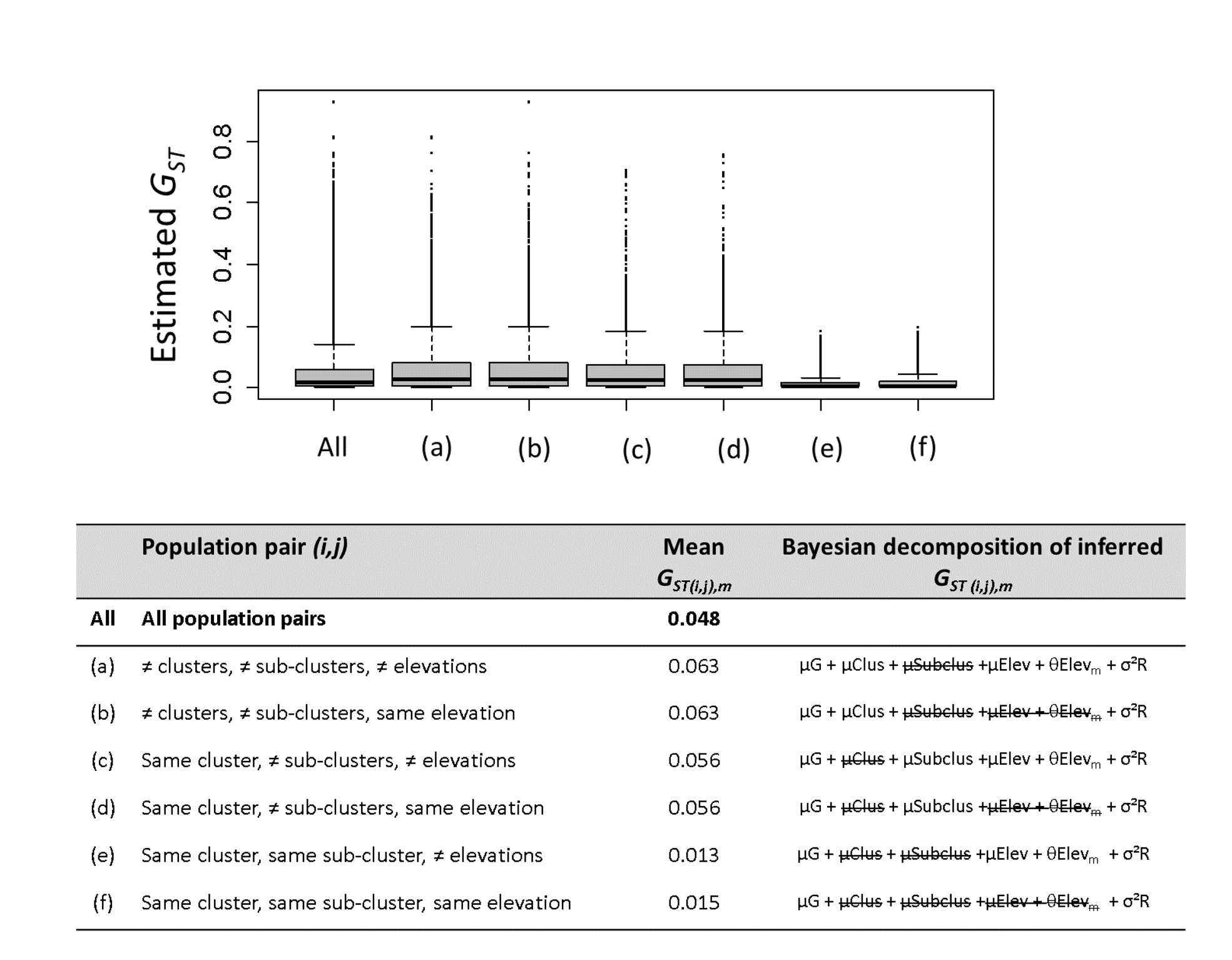

Supplement: S8 Fig — Distributions of locus-specific GST among pairs of A. alba populations (site×elevation) inferred using the hierarchical approach (HBM). Population pairs were classified depending on their membership to the same cluster (K = 2), sub-cluster (K = 4), and elevation according to the hierarchical approach, cases (a) to (f). The table included below shows the mean GST for each case (a) to (f) and details how GST values are partitioned into genome-wide and locus-specific effects by HBM. Notice that the parameters μClus, μSubClus and/or μElev are not applied when the populations (i,j) belong to the same cluster (kClus(i,j) = 0), to the same sub-cluster (kSubClus(i,j) = 0), and/or to the same elevation (kElev(i,j) = 0). (TIF) [file pone.0158216.s008.tif]

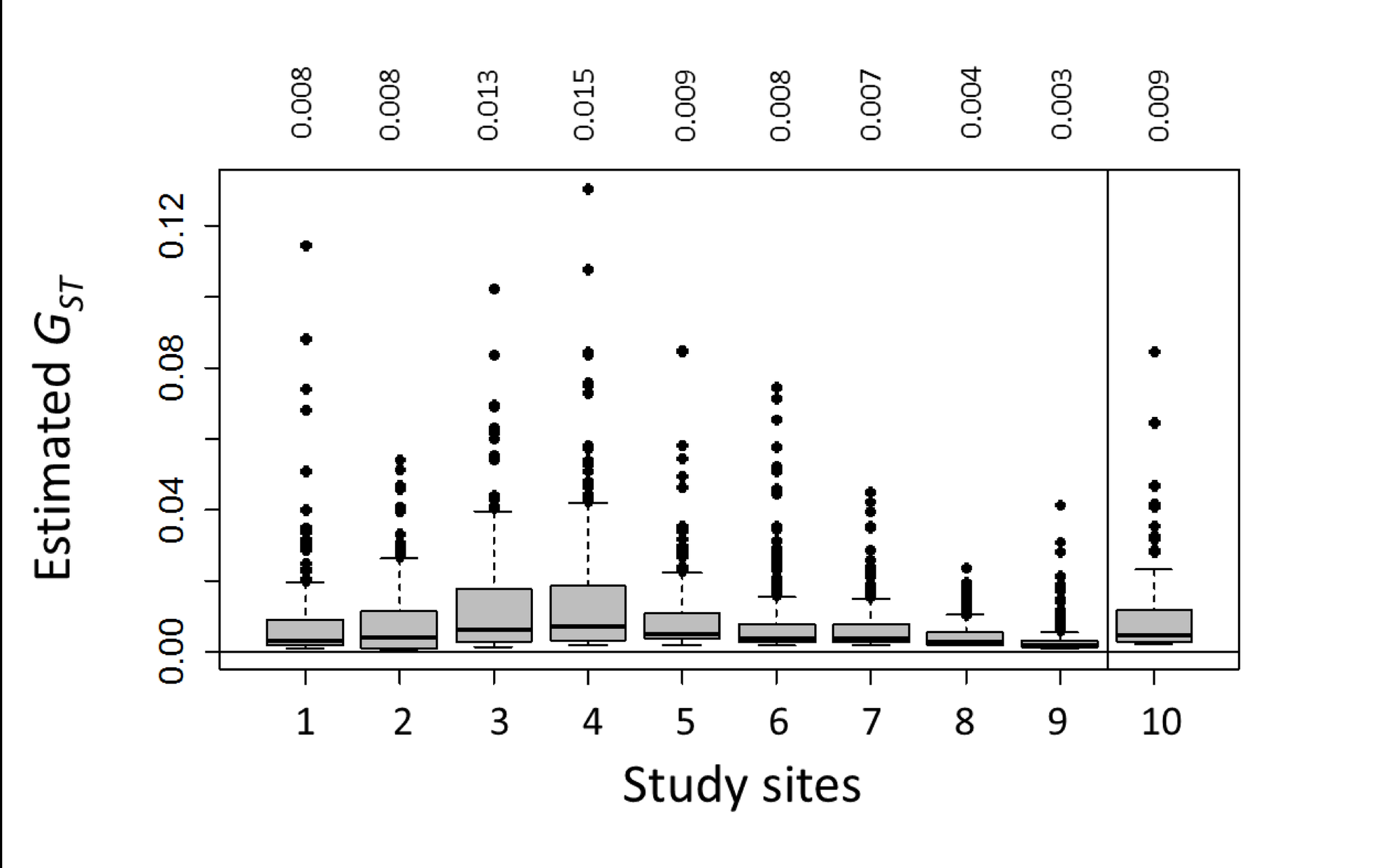

Supplement: S9 Fig — Distribution of locus-specific GST between elevations within each study site inferred through classical ‘within-site’ approach (SBM). The values above the plot show the mean differentiation among all markers in each site. Sites ID are described in Table 1. (TIF) [file pone.0158216.s009.tif]

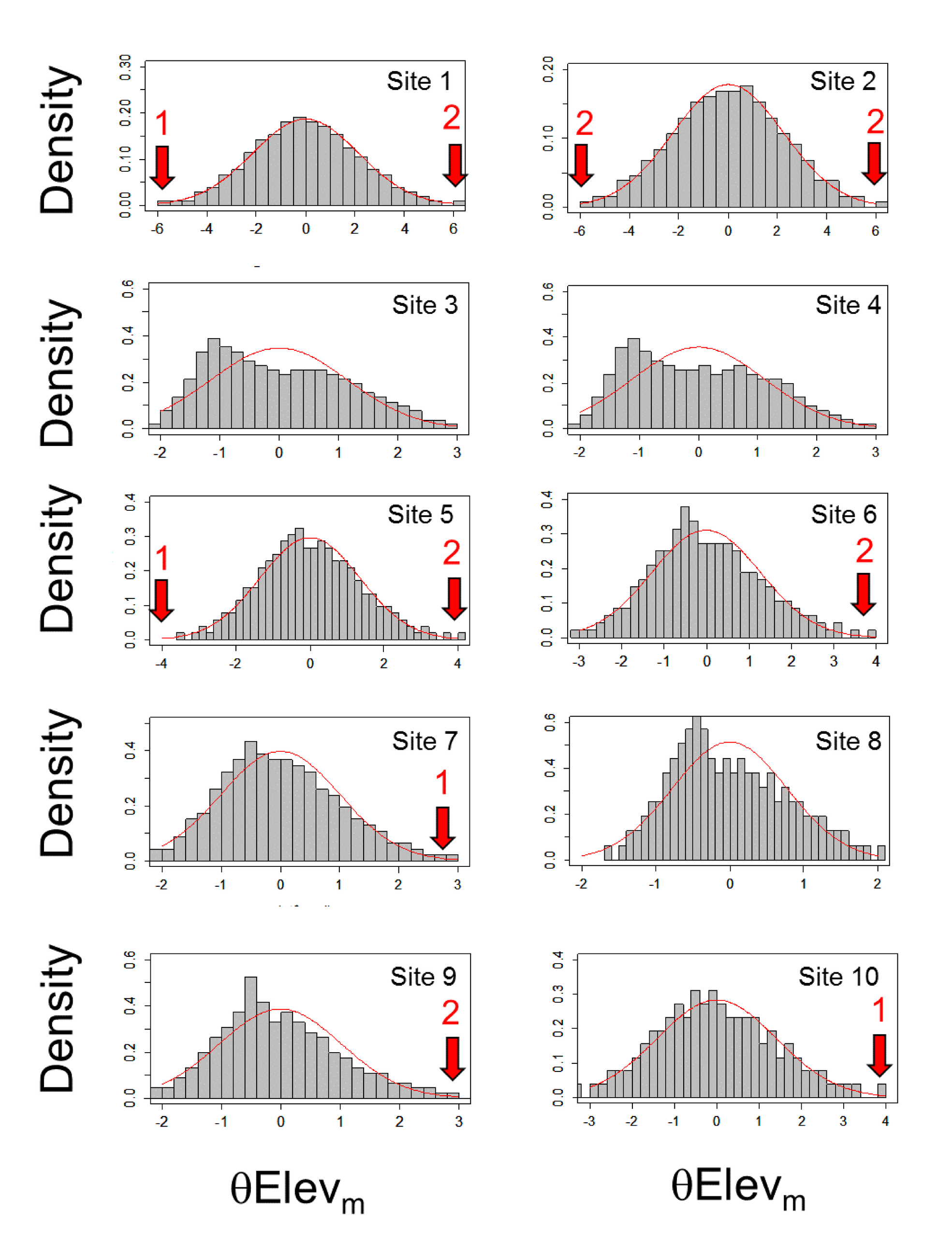

Supplement: S10 Fig — Results of SBM within each A. alba and A. cephalonica site. The arrows indicate the detected outliers for homogenizing (left-tail) and divergent (right-tail) selection under 1% threshold. Sites IDs are described in Table1. (TIF) [file pone.0158216.s010.tif]

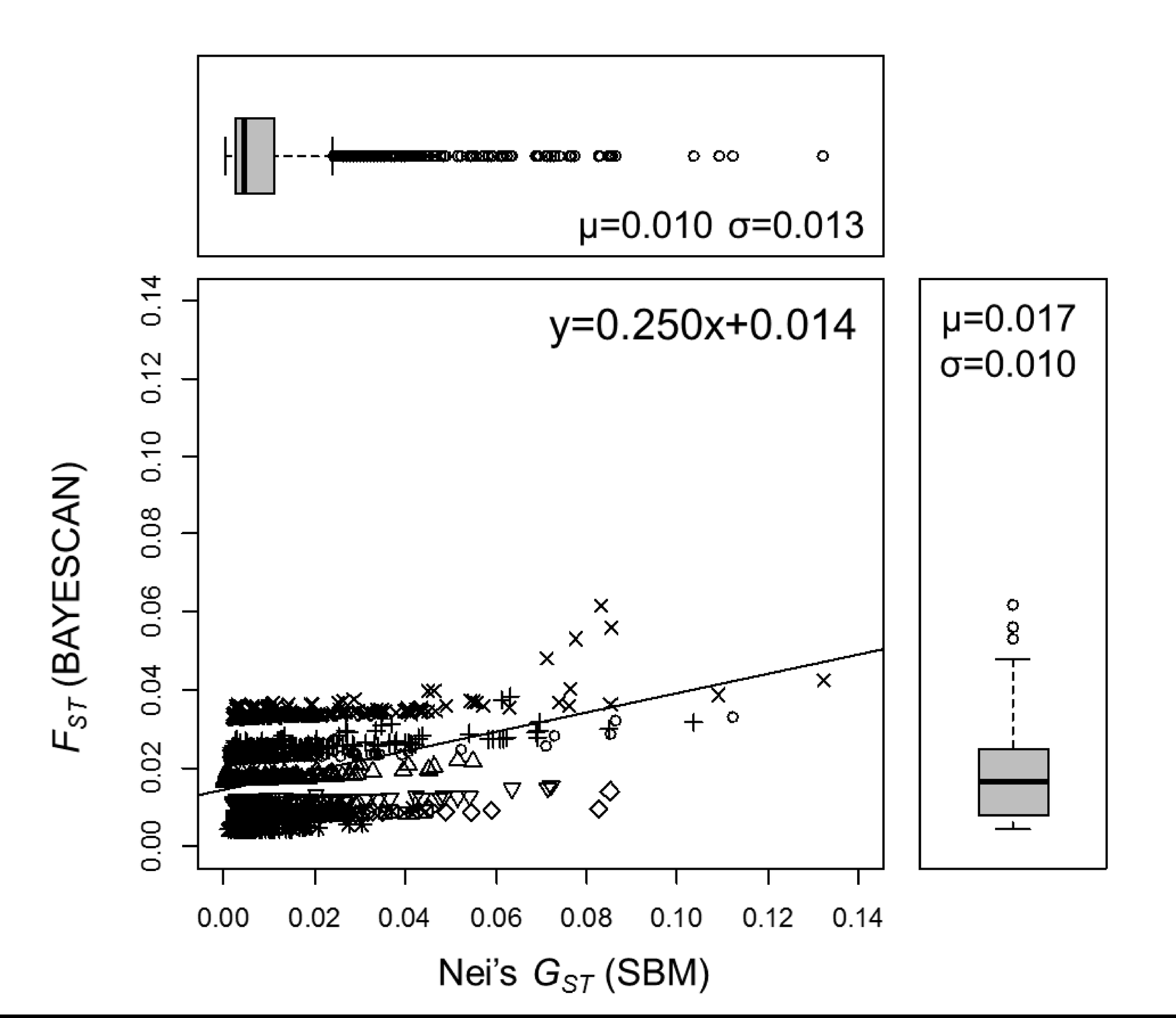

Supplement: S11 Fig — Comparison of locus-specific differentiation indices estimated within the different sites: FST is estimated using BAYESCAN and Nei’s GST is estimated using SBM. Different symbols were used for the different sites. (TIF) [file pone.0158216.s011.tif]
