# Supplementary material for: Local Adaptation in European Firs Assessed through Extensive Sampling across Altitudinal Gradients in Southern Europe
Source: PLoS One. 2016 Jul 8;11(7):e0158216. doi: 10.1371/journal.pone.0158216 (PMC4938419; doi:10.1371/journal.pone.0158216)
Supplement: S1 Method — (PDF) [file pone.0158216.s012.pdf]

Sixteen populations of size N=200 diploid individuals (*i.e.* 2n=400 alleles, that can be viewed as each *site×elevation* modality in our real data) were simulated under a hierarchical island model of migration totaling N=3200 diploid individuals (2n=6400 alleles) and assuming: two genetic clusters, each composed of two sub-clusters, with two replicated population pairs (*i.e.* two sites) within each sub-cluster and two elevations by site (Fig. S1a). One hundred bi-allelic SNPs distributed over 20 chromosomes with 5 loci per chromosome were simulated. The initial allele frequency at each locus was 0.5 and the recombination rate among loci 0.01. Five hundred generations (with random mating within populations and Mendelian transmission) were simulated under a hierarchical model of migration: $m_{inter\text{-}cluster} = 0.01 < m_{inter\text{-}subcluster} = 0.05 < m_{inter\text{-}populations} = 0.2$. Divergent and/or homogenizing selection was applied to selected loci by adding locus-specific effects that modulated parental fitness. Parental fitness (ω) expresses the probability that each parent is picked to produce offspring, with ω=1-*s* where *s* is the selection strength. When multiple loci are selected, parental fitness values were obtained by multiplying fitness across selected loci. In the case of homogenizing selection, the fitness of parental genotypes at selected loci was $\omega_{(11)}=1$, $\omega_{(12)}= [1-(s/2)]$ and $\omega_{(22)}= (1-s)$ for the genotypes (11), (12) and (22) in all simulated populations whatever their elevation. In the case of divergent selection, the fitness of parental genotypes at selected loci was $\omega_{(11)}=1$, $\omega_{(12)}= [1-(s/2)]$, and $\omega_{(22)}= (1-s)$ at one elevation, and $\omega_{(11)}= (1-s)$, $\omega_{(12)}= [1-(s/2)]$, and $\omega_{(22)}=1$ at the other elevation. An example code is available on Figshare doi: 10.6084/m9.figshare.1385219.

SBM was tested on populations pairs (*i.e.* 2 populations) made of N=1600 diploid individual each (*i.e.* 2n=3200 alleles, totaling N=3200 diploid individuals) under the same selection scenarios.
