# Supplementary material for: Local Adaptation in European Firs Assessed through Extensive Sampling across Altitudinal Gradients in Southern Europe
Source: PLoS One. 2016 Jul 8;11(7):e0158216. doi: 10.1371/journal.pone.0158216 (PMC4938419; doi:10.1371/journal.pone.0158216)
Supplement: S2 Method — R script and functions. (PDF) [file pone.0158216.s013.pdf]

This script aims at testing associations between genotypes and habitat for bi-allelic markers in study sites composed of two sub-populations (inhabiting two local habitats). The script is easily usable in this state, or can be modified at leisure by the users.

The input file *'Data_genotypes'* is a tab-delimited text file containing individual genotypes, one line by individual, one column by marker + X supplementary columns.

This table should contain at least X=2 supplementary columns containing the study site and the local habitat of each individual (at the beginning of the table). These two columns may be filled either by numeric or by a chain of characters).

- ➔ If the data consist in a unique study site, fill the column corresponding to site with "NA" (don't remove the column).
- ➔ If your first column consists in the individual identifier, add a header to that column and count it as a supplementary column.

The following '*Nmarker'* columns contain individual genotypes for the different markers in the format: AC (heterozygote A/C), AA (homozygote A/A), NA for missing values.

The input data file and all the function should be placed in a common directory.

For running the program, open R and set the different parameters using the commands:

```
workingdir="C:/Users/…"
      # working directory containing the functions and the data

inputfile="data_genotypes.txt" # input file containing the data

Nsup=5 # number of supplementary columns
Col_site=4 # index of the column containing the site information
Col_env=5# index of the column containing the local habitat
information

site="FR_Ossau"
      # name of the study site to use, as written in the "Site"
column
       # of the data file
      # Set NA to select all study sites or if the dataset
      # is composed of one study site

N1=100 # N values of F to sort in the empirical distribution of F
N2=100 # Number of samplings from the meta-population
Nind=1000 # Number of individuals to simulate in the meta-population
```

Then load the data:

```
############### Data loading
setwd(workingdir)
data=read.table(inputfile,header=T)
data=as.matrix(data)
```

You can then run the different function in the order : 1 `'convert'`, 2 `'monomorphic_rm,statistics'`, 3 `'sampling_Fis'`, 4 `'simulations'`, 5 `'associations'`.

The functions are provided at the end of this document. Copy and paste each function in a separated text file with the extention **.function**. Functions need to be sourced before running and eventually compiled through the commands:

```
source('convert.function')
library(compiler)
convert.comp=cmpfun(convert)
```

Then, the compiled function can be executed through the command

```
convert.comp(Nsup,data)
```

Here, the complete R script for running all the functions:

```
############### Conversion of the input file
convert.comp(Nsup,data)

############### Data subset and monomorphic removal (within site)
monomorphic_rm.comp(Nsup,data,site,Col_site)

############### Observed statistics
statistics.comp(Nsup,data2,Col_env)

############### Empirical distribution of F (fixation index F) and
sampling
sampling_Fis.comp(Fis,N1)

############### Expected genotypic frequencies and counts estimation
simulations.comp(Nsup,data2,Fis.sim,p,q,N1,N2,Col_env)

      #head(HT.sim)

############### empirical distribution of expected genotypic
frequencies ############### genotype-habitat associations
associations.comp(
Nsup,data2,site,
Ht.1,Ht.2,Ho1.1,Ho1.2,Ho2.1,Ho2.2,
Ht.1.sim,Ht.2.sim,Ho1.1.sim,Ho1.2.sim,Ho2.1.sim,Ho2.2.sim
)
```

The output is a tab-delimited text table of [Nmarker] lines and 6 columns. The columns contain the results of the association tests of each genotype in each local habitat type and are named '*Habitat1_Homo1*', '*Habitat1_Htz*', '*Habitat1_Homo2*', '*Habitat2_Homo1*', '*Habitat2_Htz*', '*Habitat2_Homo2*'

- ➔ The habitats are ordered in an alphabetic order, according to the name of the different habitats in the input file: Habitat1 refers to the first habitat in alphabetic order.

- ➔ The genotypes are also ordered in alphabetic order, meaning that '*Homo1'* refers to the genotype 'AA' and '*Homo2'* to 'TT' in a bi-allelic marker with alleles 'A' and 'T'. 'Htz' refers to the heterozygote genotype 'AT'.
- ➔ *'+'* means that the association is significantly positive (the genotype is more abundant than expected in that habitat), *'+'* that it is significantly negative, and '*ns*' that the association is not significant.

Here, the functions:

convert.function

```
convert=function(Nsup,data)
{
  data[is.na(data)]=""

  data.new=matrix(NA,nrow=nrow(data),ncol=ncol(data))

  colnames(data.new)=colnames(data)
  data.new[,(1:Nsup)]=as.matrix(data[,(1:Nsup)])

  for (i in 1:nrow(data))
  {
    for (j in (Nsup+1):ncol(data))
    {
      if(data[i,j]!="")
      {
        genot=unlist(strsplit(as.character(data[i,j]),split=""))
        genot=genot[order(genot)]
        genot=paste(genot,sep="",collapse="")
        data.new[i,j]=genot
      }else{
        data.new[i,j]=NA
      }
    }
  }
  data.new<<-data.new
}
```

Monomorphic_rm.function

```
monomorphic_rm =function(Nsup,data.new,site,Col_site){

data=data.frame(data.new)

if(is.na(site)==F){
data2=data[which(data[,Col_site]==site),]
}else{
data2=data
}

remove=vector()
for (j in (Nsup+1):ncol(data2))
{
```

```
remove[j]=ifelse(length(levels(as.factor(as.vector(data2[,j]))))==1,
1,0)
}
if(length(which(remove==1))>0)
{
data2=data2[,-which(remove==1)]
}

# missing markers removal
remove=vector()
for (j in (Nsup+1):ncol(data2))
{

remove[j]=ifelse(length(which(is.na(data2[,j])))==length(data2[,j]),
1,0)
}

if(length(which(remove==1))>0)
{
data2=data2[,-which(remove==1)]
}

data2<<-data2

}
```

statistics.function

```
statistics=function(Nsup,data2,Col_env)
{

p=vector()
q=vector()
HT=vector()
Ht.1=vector()
Ht.2=vector()
Ho1.1=vector()
Ho1.2=vector()
Ho2.1=vector()
Ho2.2=vector()
Fis=vector()

all_env=levels(as.factor(data2[,Col_env]))

  for (j in (Nsup+1):ncol(data2))
  {
    genotypes=as.factor(data2[,j])
    genot=levels(genotypes)

    genotypes.2=genotypes
    genotypes.2=as.matrix(genotypes.2)
    genotypes.2[is.na(genotypes.2)]=""

    alleles=unlist(strsplit(as.character(genotypes.2),split=""))
    all=levels(as.factor(unique(alleles)))
```

```
    htz=paste(all,sep="",collapse="")
    h1=paste(all[1],all[1],sep="",collapse="")
    h2=paste(all[2],all[2],sep="",collapse="")

    # in the whole population

p[j]=length(which(alleles==all[1]))/length(which(is.na(alleles)==F))

q[j]=length(which(alleles==all[2]))/length(which(is.na(alleles)==F))

HT[j]=length(which(genotypes==htz))/length(which(is.na(genotypes)==F
))
    Fis[j]=1-(HT[j]/(2*p[j]*q[j]))

    # by population
    genotypes_pop1=genotypes[which(data2[,Col_env]==all_env[1])]
    genotypes_pop2=genotypes[which(data2[,Col_env]==all_env[1])]
#pop1=high
#pop2=low

Ht.1[j]=length(which(genotypes_pop1==htz))/length(which(is.na(genoty
pes_pop1)==F))

Ht.2[j]=length(which(genotypes_pop2==htz))/length(which(is.na(genoty
pes_pop2)==F))


Ho1.1[j]=length(which(genotypes_pop1==h1))/length(which(is.na(genoty
pes_pop1)==F))

Ho2.1[j]=length(which(genotypes_pop1==h2))/length(which(is.na(genoty
pes_pop1)==F))


Ho1.2[j]=length(which(genotypes_pop2==h1))/length(which(is.na(genoty
pes_pop2)==F))

Ho2.2[j]=length(which(genotypes_pop2==h2))/length(which(is.na(genoty
pes_pop2)==F))

   }
  p<<-p
  q<<-q
  HT<<-HT
  Ht.1<<-Ht.1
  Ht.2<<-Ht.2
  Ho1.1<<-Ho1.1
  Ho2.1<<-Ho2.1
  Ho1.2<<-Ho1.2
  Ho2.2<<-Ho2.2
  Fis<<-Fis

}
```

sampling_Fis.function

```
sampling_Fis=function(Fis,N1)
{

fit=density(Fis[-which(is.na(Fis))])
bw <- fit$bw

par(mfrow=c(2,1),mar=c(1,2,4,1))
hist(Fis,nclass=50,prob=T,col="grey",xlim=c(-1,1),ylim=c(0,10))
lines(fit)

# Draw from the sample and then from the kernel
means <- sample(Fis[-which(is.na(Fis))], N1, replace = TRUE)
sample.fis=rnorm(N1, mean = means, sd = bw)
hist(sample.fis, prob = TRUE,nclass=20,col="grey",xlim=c(-
1,1),ylim=c(0,10))
#lines(fit)

Fis.sim<<-sample.fis

Fis.mean<<-mean(Fis,na.rm=T)

}
```

simulations.function

```
simulations=function(Nsup,data2,Fis.sim,p,q,N1,N2,Col_env)
{

FIS.sim=matrix(NA,nrow=N1*N2,ncol=ncol(data2))
HT.sim=matrix(NA,nrow=N1*N2,ncol=ncol(data2))
Hexp.tot.sim=matrix(NA,nrow=N1*N2,ncol=ncol(data2))

Ht.1.sim=matrix(NA,nrow=N1*N2,ncol=ncol(data2))
Ht.2.sim=matrix(NA,nrow=N1*N2,ncol=ncol(data2))
Ho1.1.sim=matrix(NA,nrow=N1*N2,ncol=ncol(data2))
Ho1.2.sim=matrix(NA,nrow=N1*N2,ncol=ncol(data2))
Ho2.1.sim=matrix(NA,nrow=N1*N2,ncol=ncol(data2))
Ho2.2.sim=matrix(NA,nrow=N1*N2,ncol=ncol(data2))

all_env=levels(as.factor(data2[,Col_env]))

  for (j in (Nsup+1):ncol(data2)) # for each marker
  {
    x=0


    genotypes=as.factor(data2[,j])
    genotypes_pop1=genotypes[which(data2[,Col_env]==all_env[1])]
    genotypes_pop2=genotypes[which(data2[,Col_env]==all_env[2])]
#pop1 =high
#pop1 =low
    N.1=length(which(is.na(genotypes_pop1)==F))
    N.2=length(which(is.na(genotypes_pop2)==F))
```

```
    for (n1 in 1:N1) # for each simulated Fis
    {

      # in the wole population
      Ht.sim.temp=(2*p[j]*q[j])*(1-Fis.sim[n1])
      H1.sim.temp=(p[j])^2+(p[j]*q[j]*Fis.sim[n1])
      H2.sim.temp=(q[j])^2+(p[j]*q[j]*Fis.sim[n1])

      # indiviuals counts at the whole pop level
      Nt.sim=round(Nind*Ht.sim.temp)
      N1.sim=round(Nind*H1.sim.temp)
      N2.sim=round(Nind*H2.sim.temp)

      if(Nt.sim<0){Nt.sim=0}
      if(N1.sim<0){N1.sim=0}
      if(N2.sim<0){N2.sim=0}

      Nt=Nt.sim+N1.sim+N2.sim
      All.ind=c(rep("ht",Nt.sim),rep("h1",N1.sim),rep("h2",N2.sim))


      for (n2 in 1:N2) # sampling between subpopulations
      {
        x=x+1

        pop1.sim=sample(All.ind,500)
        pop2.sim=sample(All.ind,500)
        # each population is a sample from pool of individual of the
metapop not structured

        Ht.1.sim[x,j]<<-
length(which(pop1.sim=="ht"))/length(pop1.sim)
        Ht.2.sim[x,j]<<-
length(which(pop1.sim=="ht"))/length(pop2.sim)

        Ho1.1.sim[x,j]<<-
length(which(pop1.sim=="h1"))/length(pop1.sim)
        Ho2.1.sim[x,j]<<-
length(which(pop1.sim=="h2"))/length(pop1.sim)

        Ho1.2.sim[x,j]<<-
length(which(pop2.sim=="h1"))/length(pop2.sim)
        Ho2.2.sim[x,j]<<-
length(which(pop2.sim=="h2"))/length(pop2.sim)


        HT.sim[x,j]<<-Ht.sim.temp
        FIS.sim[x,j]<<-Fis.sim[n1]


      }
    }
  }
}
```

associations.function

```
associations=function(
Nsup,data2,site,
Ht.1,Ht.2,Ho1.1,Ho1.2,Ho2.1,Ho2.2,
Ht.1.sim,Ht.2.sim,Ho1.1.sim,Ho1.2.sim,Ho2.1.sim,Ho2.2.sim
)
{
#pop1 =high
#pop2 =low

Genotypes_proba=matrix(NA,nrow=ncol(data2),ncol=6)
colnames(Genotypes_proba)=c("Habitat1_homo1","Habitat1_htz","Habitat
1_homo2","Habitat2_homo1","Habitat2_htz","Habitat2_homo2")

for (j in (Nsup+1):ncol(data2)) # for each marker
{
  #for Htz in habitat 1 (high)
  dens=density(Ht.1.sim[,j],n=1000,from=0,to=1)
  cumprob=cumsum(dens$y)
  cumprob.std=cumprob/max(cumprob)
  #plot(dens$x,cumprob.std)
  probas=cumprob.std[which(round(dens$x,2)==round(Ht.1[j],2))]
  proba=mean(probas)
  Genotypes_proba[j,5]=proba

  #for Htz in habitat 2 (low)
  dens=density(Ht.2.sim[,j],n=1000,from=0,to=1)
  cumprob=cumsum(dens$y)
  cumprob.std=cumprob/max(cumprob)
  probas=cumprob.std[which(round(dens$x,2)==round(Ht.2[j],2))]
  proba=mean(probas)
  Genotypes_proba[j,2]=proba

  #for Homo1 in habitat 1 (high)
  dens=density(Ho1.1.sim[,j],n=1000,from=0,to=1)
  cumprob=cumsum(dens$y)
  cumprob.std=cumprob/max(cumprob)
  probas=cumprob.std[which(round(dens$x,2)==round(Ho1.1[j],2))]
  proba=mean(probas)
  Genotypes_proba[j,4]=proba

  #for Homo1 in habitat 2 (low)
  dens=density(Ho1.2.sim[,j],n=1000,from=0,to=1)
  cumprob=cumsum(dens$y)
  cumprob.std=cumprob/max(cumprob)
  probas=cumprob.std[which(round(dens$x,2)==round(Ho1.2[j],2))]
  proba=mean(probas)
  Genotypes_proba[j,1]=proba


  # for j=5, j=10
  #x11()
  #plot(dens,xlim=c(0.9,1),col="blue")
  #abline(v=Ho1.2[j],col="red")
```

```
  #plot(dens$x,cumprob.std,xlim=c(0.9,1),type="l",col="blue")
  #abline(v=Ho1.2[j],col="red")

  #observed genotypic frequencies
  #table(data2[,Col_env],data2[,j])
  #AA AT TT
  #high 47  1  0
  #low  41  2  0

  # resus association test
  #AA AT TT
  #high ns ns ns
  #low  -  +  ns

  #for Homo2 in habitat 1 (high)
  dens=density(Ho2.1.sim[,j],n=1000,from=0,to=1)
  cumprob=cumsum(dens$y)
  cumprob.std=cumprob/max(cumprob)
  probas=cumprob.std[which(round(dens$x,2)==round(Ho2.1[j],2))]
  proba=mean(probas)
  Genotypes_proba[j,6]=proba

  #for Homo2 in habitat 2 (low)
  dens=density(Ho2.2.sim[,j],n=1000,from=0,to=1)
  cumprob=cumsum(dens$y)
  cumprob.std=cumprob/max(cumprob)
  probas=cumprob.std[which(round(dens$x,2)==round(Ho2.2[j],2))]
  proba=mean(probas)
  Genotypes_proba[j,3]=proba

}

Genotypes_proba.clean=Genotypes_proba
Genotypes_proba.clean[Genotypes_proba.clean<=0.01]="-"
Genotypes_proba.clean[Genotypes_proba.clean>=0.99]="+"
Genotypes_proba.clean[(Genotypes_proba.clean>0.01)&(Genotypes_proba.
clean<0.99)]="ns"

rownames(Genotypes_proba.clean)=colnames(data2)
Genotypes_proba.clean=Genotypes_proba.clean[-(1:Nsup),]

#fix(Genotypes_proba.clean)

test=as.vector(Genotypes_proba.clean)
table(test)

# number of significant and not significant associations
#-    +   ns
#138  119 1003
# the simulations reveal well the reality, as the majority of
associations # are ns !!!


#fix(Genotypes_proba.clean)
#fix(Genotypes_proba)
```

```
outfile=paste("output_",site,".txt",sep="",collapse="")
write.table(Genotypes_proba.clean,outfile,sep="\t",row.names=T,col.names=T,quote=F)

}
```
