# Supplementary material for: Local Adaptation in European Firs Assessed through Extensive Sampling across Altitudinal Gradients in Southern Europe
Source: PLoS One. 2016 Jul 8;11(7):e0158216. doi: 10.1371/journal.pone.0158216 (PMC4938419; doi:10.1371/journal.pone.0158216)
Supplement: S1 Table — Outliers are sorted by their position relative to the neutral background: I. Outliers detected above the neutral background; II. Outliers detected above the neutral background in one site and below the neutral background in another site; III. Outliers detected below the neutral background. The first column describes the SNP number, the second column the SNP ID. The third column describes the study site in which the outliers were detected and the method used: (a) HBM under a 1% threshold (hierarchical multi-site Bayesian method), (b) FDIST (within-site coalescent method), (c) BAYESCAN (within-site Bayesian method), (d) SBM under a 1% threshold (within-site Bayesian method). Study sites IDs are described in Table 1. The 12 outliers detected twice above the neutral background (by two different approaches) are shaded in grey. (PDF) [file pone.0158216.s014.pdf]

SNP N°	SNP ID	Study site ID (method)
**I. Outliers detected above the neutral background**		
10	contig00945.39	Site 4 (b)
15	contig01131.558	Site 4 (b)
29	contig02088.183	Site 1 (b),(d)
32	contig02161.64	Site 10 (b)
50	contig02986.327	Site 4 (b)
58	contig03942.73	Site 9 (b),(d)
61	contig04538.344	Site 6 (b),(d)
76	contig06119.125	Site 3 (b)
84	contig06968.51	Site 6 (b),(d)
102	contig08855.98	Site 3 (b) / Site 6 (b)
110	contig10568.484	Site 9 (b)
113	contig11291.4439	Site 2 (b),(d) / Site 7 (b)
139	contig15135.274	Site 4 (b)
140	contig15135.373	Site 4 (b)
153	contig15663.771	Site 3 (b)
157	contig16125.157	Site 5 (b),(d)
158	contig16125.585	Site 4 (b)
161	contig16332.419	Site 1 (b) / Site 10 (b),(d)
203	contig20694.1090	Site 7 (b),(d)
210	contig23982.493	Site 6 (b)
230	contig31121.47	Site 6 (b)
236	contig01181.99	Site 3 (b)
253	contig08206.176	Site 1 (b)
255	contig09373.367	Site 9 (b),(d)
265	contig20680.335	Site 7 (b)
**II. Outliers detected above the neutral background in one site and below the neutral background in another site**		
65	contig05004.249	Site 1, above (b),(d) / Site 2, below (b)
99	contig08649.617	Site 4, below (b) / Site 5, above (b),(d)
170	contig16774.460	Site 1, above (b) / Site 4, below (b)
176	contig17776.918	Site 4, above (b) / Site 9, below (b)
258	contig15452.813	Site 2, above (b),(d) / Site 6, below (b)
**III. Outliers detected below the neutral background**		
1	contig00241.160	Site 9 (b)
2	contig00241.632	Site 5 (b)
3	contig00509.93	Site 1(b) / Site 10 (b)
23	contig01505.255	Site 9 (b)
24	contig01505.256	Site 1(b)
26	contig02019.588	ALL (a)
34	contig02190.239	Site 9 (b)
43	contig02676.141	Site 5 (b) / Site 6 (b)
51	contig03380.79	Site 1 (b)
56	contig03803.208	Site 1 (b) / Site 2 (d)
63	contig04911.323	Site 7 (b) / Site 9 (b)
69	contig05306.661	Site 2 (d)
79	contig06641.50	Site 2 (b)
81	contig06757.154	Site 2 (b)
90	contig07377.313	Site 6 (b) / Site 9 (b)
94	contig08213.555	Site 6 (b)
101	contig08815.372	Site 2 (b)
104	contig09501.1038	Site 5 (b)

111	contig10976.962	ALL[(a)]
121	contig14310.698	Site 2 [(b)]
124	contig14409.108	Site 6 [(b)]
125	contig14455.629	Site 2 [(b)]
132	contig14680.474	Site 10 [(b)]
142	contig15196.214	Site 7 [(b)]
149	contig15484.725	Site 6 [(b)]
155	contig15808.829	Site 9 [(b)]
162	contig16356.332	Site 2 [(b)] / Site 5 [(b)]
163	contig16411.197	Site 9 [(b)]
165	contig16454.530	Site 1 [(b)] / Site 9 [(b)]
168	contig16756.89	Site 6 [(b)]
173	contig16884.608	Site 10 [(b)]
178	contig17813.614	Site 2 [(b)]
182	contig18599.280	Site 5 [(b)]
183	contig18599.772	Site 3 [(b)]
184	contig18599.823	Site 2 [(b)]
187	contig18676.245	Site 9 [(b)]
192	contig19173.687	Site 1 [(b)] / Site 2 [(b)]
194	contig19232.367	Site 1 [(d)]
196	contig19448.666	Site 3 [(b)]
199	contig20262.932	Site 9 [(b)]
201	contig20694.92	Site 2 [(b)]
204	contig21068.129	Site 10 [(b)]
218	contig24902.464	Site 9 [(b)]
223	contig26089.889	Site 6 [(b)] / Site 9 [(b)]
228	contig27000.1448	Site 9 [(b)]
232	contig00575.206	Site 1 [(b)]
234	contig01181.201	Site 5 [(d)]
235	contig01181.592	Site 9 [(b)]
249	contig06676.165	Site 9 [(b)]
250	contig07067.521	Site 2 [(b)]
251	contig07067.53	Site 1 [(b)]
252	contig08200.613	Site 9 [(b)]
261	contig16430.504	Site 9 [(b)]
267	contig25086.1240	Site 9 [(b)]
269	contig26602.432	Site 6 [(b)]
272	contig30715.591	Site 4 [(b)]
