# Supplementary material for: Local Adaptation in European Firs Assessed through Extensive Sampling across Altitudinal Gradients in Southern Europe
Source: PLoS One. 2016 Jul 8;11(7):e0158216. doi: 10.1371/journal.pone.0158216 (PMC4938419; doi:10.1371/journal.pone.0158216)
Supplement: S2 Table — The first column describes the SNP number, the second column the SNP ID, the third column describes the study site in which the SNP was detected as outlier for divergent selection (study sites IDs are described in Table 1) and the fourth column describes the alleles genotyped. The columns 5 to 10 describes the results of GEAs at low (columns 5 to 7) and high elevations (columns 8 to 10) for each genotype (homozygotes and heterozygote): ‘Homozygote 1’ is the first homozygote and ‘Homozygote 2’ is the second homozygote (for e.g.: for two alleles (C) and (T), (CC) is the homozygote 1 and (TT) the homozygote 2); ‘+’ indicates a positive association, ‘-’ indicates a negative association, ‘ns’ a non-significant association, and ‘NA’ a missing value (because the SNP is monomorphic in the study site. (PDF) [file pone.0158216.s015.pdf]

				Low elevation			High elevation		
SNP N°	SNP ID	Study site	Alleles	Homozygote 1	Heterozygote	Homozygote 2	Homozygote 1	Heterozygote	Homozygote 2
29	contig02088.183	1	C/T	-	ns	ns	+	ns	-
58	contig03942.73	9	G/T	ns	ns	-	-	ns	ns
61	contig04538.344	6	C/T	+	ns	-	ns	-	+
65	contig05004.249	1	C/T	-	ns	+	ns	+	-
84	contig06968.51	6	G/T	-	ns	+	+	-	ns
99	contig08649.617	5	A/G	+	ns	-	-	ns	ns
113	contig11291.4439	2	C/G	-	ns	+	+	ns	-
157	contig16125.157	5	A/G	ns	ns	+	ns	+	-
161	contig16332.419	10	G/T	ns	-	+	ns	+	-
203	contig20694.1090	7	C/T	ns	ns	ns	+	ns	ns
255	contig09373.367	9	C/T	-	+	ns	+	ns	-
258	contig15452.813	2	G/T	+	+	-	-	-	+
