# Supplementary material for: Local Adaptation in European Firs Assessed through Extensive Sampling across Altitudinal Gradients in Southern Europe
Source: PLoS One. 2016 Jul 8;11(7):e0158216. doi: 10.1371/journal.pone.0158216 (PMC4938419; doi:10.1371/journal.pone.0158216)
Supplement: S3 Table — (PDF) [file pone.0158216.s016.pdf]

Study site ID	Number of polymorphic SNPs	Number of consistent outliers	Proportion of consistent outliers
1	210	2	0.95%
2	262	2	0.76%
3	257	0	0%
4	253	0	0%
5	262	2	0.76%
6	238	2	0.84%
7	233	1	0.43%
8	158	0	0%
9	228	2	0.88%
10	129	1	0.78%
