# Supplementary material for: Local Adaptation in European Firs Assessed through Extensive Sampling across Altitudinal Gradients in Southern Europe
Source: PLoS One. 2016 Jul 8;11(7):e0158216. doi: 10.1371/journal.pone.0158216 (PMC4938419; doi:10.1371/journal.pone.0158216)
Supplement: S4 Table — (PDF) [file pone.0158216.s017.pdf]

Contig name	Contig size (bp)	SNP(s) number	SNP(s) Position within contig (base)	BlastX	eValue	GO terms (biological processes levels3 & 4)	KEGG enzyme code	Additional comment
contig02088	507	29	183	polyubiquitin 4-partial	6.15E-70	Level 3 : catabolic process, cellular metabolic processs, **primary metabolic process**, organic substance metabolic process<br>Level 4 : cellular catabolic process, macromolecule metabolic process, organic substance catabolic process	-	
contig03942	478	58	73	unknown	1.54E-65	Level 3: catabolic process, cellular metabolic process, **primary metabolic process**, organic substance metabolic process<br>Level 4 : cellular catabolic process, macromolecule metabolic process, organic substance catabolic process	EC:3.4.25	
contig04538	557	61	344	unknown	4.95E-53	unknown	-	
contig05004	779	65	249	magnesium chelatase h-like protein	3.39E-116	Level 3: **nitrogen compound metabolic process**, cellular metabolic<br>Level 4: **pigment metabolic process**, **photosynthesis**, **cellular nitrogen compound metabolic process**, cellular aromatic compound metabolic process, organic substance biosynthetic process, heterocycle metabolic process, cofactor metabolic process, generation of precursor metabolites and energy, organic cyclic compound metabolic process, cellular biosynthetic process, organonitrogen compound metabolic process	EC:6.6.1.1	first committed step of chlorophyll biosynthesis and a branchpoint of two major routes in the tetrapyrrole pathway
contig06968	418	84	51	eukaryotic peptide chain release factor subunit 1-2 family protein	1.08E-87	Level 3: cellular metabolic process, **primary metabolic process**, single-organism cellular process, cellular component organization, organic substance metabolic process, biosynthetic process<br>Level 4: macromolecule metabolic process, organic substance biosynthetic process, macromolecular complex subunit organization, cellular component disassembly, cellular	-	
contig08649	769	99	617	ethylene-responsive transcription factor-like protein at4g13040		Level 3: **nitrogen compound metabolic process**, cellular metabolic<br>Level 4: regulation of metabolic process, macromolecule metabolic	-	

contig09373	1297	255	367	lim2 transcription factor	3.36E-133		-	
contig11291	2340	113	4439	membrane-bound endo- -beta-glucanase	0	Level 3: **primary metabolic process**, organic substance metabolic process<br>Level 4: carbohydrate metabolic process	EC:3.2.1.4	Carbohydrate metabolic processes, including the formation of carbohydrates derivatives
contig15452	1222	258	813	glutamine synthetase	0	Level 3: **nitrogen compound metabolic process**, cellular metabolic<br>Level 4: organic substance biosynthetic process, small molecule	EC:6.3.1.2	
contig16125	676	157	157	tpa: ubiquitin fusion protein	4.06E-92	Level 3: catabolic process, cellular metabolic process, **primary metabolic process**, organic substance metabolic process<br>Level 4: cellular catabolic process, macromolecule metabolic process, organic substance catabolic process	-	Membrane ubiquitin-dependent protein involved in the breakdown of proteins or peptide
contig16332	572	161	419	n-terminal nucleophile aminohydrolases (ntn hydrolases)	5.04E-88	Level 3: catabolic process, cellular metabolic process, **primary metabolic process**, organic substance metabolic process<br>Level 4: cellular catabolic process, macromolecule metabolic	EC:3.4.25	
contig20694	1563	203	1090	protein	0	Level 3: single-organism cellular process, cellular component organization<br>Level 4: cell wall organization or biogenesis, external encapsulating structure organization	EC:3.1.1.11	
